# CXCR4 knockdown enhances sensitivity of paclitaxel via the PI3K/Akt/mTOR pathway in ovarian carcinoma

**DOI:** 10.18632/aging.203241

**Published:** 2022-06-09

**Authors:** Dan Zi, Qing Li, Cheng-xiong Xu, Zhi-Wei Zhou, Guan-Bin Song, Cheng-Bin Hu, Fang Wen, Han-Lin Yang, Lei Nie, Xing Zhao, Jun Tan, Shu-Feng Zhou, Zhi-Xu He

**Affiliations:** 1 Department of Obstetrics and Gynecology, Guizhou Provincial People’s Hospital, Guiyang 550002, Guizhou, China; 2 Department of Obstetrics and Gynecology, Affiliated Hospital of Guizhou Medical University, Guiyang 550004, China; 3 Key Laboratory of Adult Stem Cell Transformation Research, Chinese Academy of Medical Sciences/Stem Cell and Tissue Engineering Research Center, Guizhou Medical University, Guiyang 550004, China; 4 Key Laboratory of Endemic and Ethnic Diseases and Key Laboratory of Molecular Biology, Ministry of Education, Guizhou Medical University, Guiyang 550004, China; 5 Department of Pharmaceutical Sciences, College of Pharmacy, University of South Florida, Tampa, FL 33612, USA; 6 Cancer Center, Daping Hospital and Research Institute of Surgery, The Third Military Medical University, Yuzhong 40042, Chongqing, China; 7 Department of Radiation Oncology, University of Texas Southwestern Medical Center, Dallas, TX 75390, USA; 8 Key Laboratory of Biorheological Science and Technology, Ministry of Education, College of Bioengineering, Chongqing University, Chongqing 400030, China; 9 Department of Computer Science and Engineering, University of South Florida, Tampa, FL 33620, USA; 10 Department of Bioengineering and Biotechnology, College of Chemical Engineering, Huaqiao University, Xiamen 361021, Fujian, China; 11 Department of Pediatrics, Affiliated Hospital of Zunyi Medical University, Zunyi 563000, China

**Keywords:** CXCR4, PI3K/Akt/mTOR, CSCs, ovarian cancer, PTX

## Abstract

Epithelial ovarian cancer (EOC) is the deadliest gynecological malignancy. EOC control remains difficult, and EOC patients show poor prognosis regarding metastasis and chemotherapy resistance. The aim of this study was to estimate the effect of CXCR4 knockdown-mediated reduction of cancer stem cells (CSCs) and epithelial–mesenchymal transition (EMT) stemness and enhancement of chemotherapy sensitivity in EOC. Mechanisms contributing to these effects were also explored. Our data showed distinct contribution of CXCR4 overexpression by dependent PI3K/Akt/mTOR signaling pathway in EOC development. CXCR4 knockdown resulted in a reduction in CSCs and EMT formation and enhancement of chemotherapy sensitivity in tumor cells, which was further advanced by blocking CXCR4-PI3K/Akt/mTOR signaling. This study also documented the critical role of silencing CXCR4 in sensitizing ovarian CSCs to chemotherapy. Thus, targeting CXCR4 to suppress EOC progression, specifically in combination with paclitaxel (PTX) treatment, may have clinical application value.

## INTRODUCTION

Among gynecological malignancies, epithelial ovarian carcinoma (EOC) is well known for its significant fatality rate, and it is also the fourth most common cause of death in women [1]. Despite recent advances in surgery and chemotherapy, the survival rate in EOC remains low due to the development of drug resistance and relapse, which are not well controlled by the current standard therapy [2]. Furthermore, there has been no important breakthrough in the control of EOC to date [3, 4]. Hence, understanding its molecular pathogenesis is vital for providing additional targeted treatments.

In the tumor microenvironment, chemokines and chemokine receptors play a critical role in tumorigenesis and metastasis. Cancer metastasis may be prompted by chemokines and their receptors. CXCR4 is the receptor for SDF-1 [5–9]; this member of the G-protein-coupled receptor (GPCR) family has seven transmembrane domains and plays a key role in lymph node and distant metastasis as well as cell migration in several types of cancer [10, 11]. CXCR4 can serve as an independent prognostic factor [12–15] in ovarian cancer, revealing a close relationship between the chemokine axis CXCL12/CXCR4 and ovarian malignancies, which has been reported in many studies. Nevertheless, the precise mechanism underlying the downstream regulation of SDF-1/CXCR4 in EOC progression is unknown. The PI3K/Akt/mTOR pathway plays a significant role in a variety of cellular processes, including cell migration, invasion, adhesion, metabolism, proliferation, and survival [16]. Phosphorylation of PI3K induced by microenvironment stimuli, including cytokines, growth factors, and hormones, accounts for Akt activation, which leads to mTOR phosphorylation and PI3K/Akt/mTOR signaling pathway activation [17–20].

It has been shown that activation of the PI3K/Akt/mTOR pathway is connected to tumor aggressiveness through the regulation of multiple cellular pathways, including the induction of CSC and EMT phenotypes, which may also be increased in many tumors [21, 22]. The previous studies have reported that CXCR4 promotes tumor progression by activating the PI3K/Akt/mTOR pathway in several types of cancer [5, 23, 24]. Thus, PI3K/Akt/mTOR pathway activation may be critically involved in CXCR4-mediated promotion of tumor aggressiveness in EOC. Our research demonstrates the impact of CXCR4 silencing in attenuating EMT and characteristics of CSC stemness as well as enhancing the sensitivity of ovarian cancer cells to PTX treatment *in vitro* and *in vivo*. Most importantly, these effects were synergistically boosted by blocking the PI3K/Akt/mTOR signaling pathway but counteracted by overexpressing CXCR4.

## RESULTS

### Clinicopathological observation indicates a clinically meaningful correlation of CXCR4 expression with ovarian cancer aggressiveness

**Figure 1:**
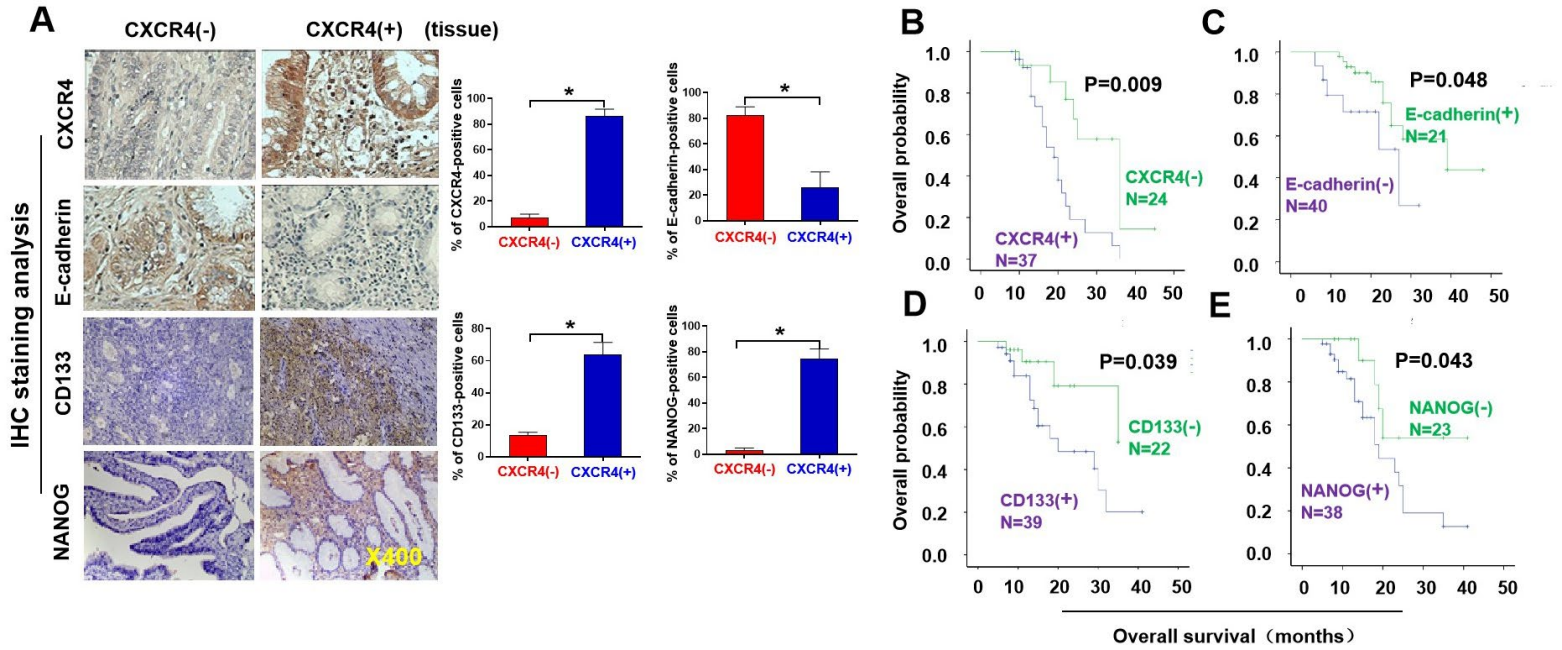
**Pathological and clinical prognostic analyses of CXCR4, EMT-and CSC-related protein expressions in epithelial ovarian cancer patients.** (**A**) Expressions of CXCR4 and E-cadherin, N-cadherin, CD133, and NANOG in EOC and benign epithelial ovarian tumour tissues were analysed by immunohistochemistry (IHC) staining with the indicated antibody against each protein examined. Notably, presence of CXCR4 expression is in epithelial ovarian cancer (EOC) tissues (mainly located in cytoplasm), but absence of CXCR4 expression in benign epithelial ovarian tumour tissues. E-cadherin expression was higher in CXCR4-negative EOC tissues than in CXCR4-positive samples. Tumour cells in the CXCR4-negative section did not express N-cadherin or vimentin, whereas some tumour cells in the CXCR4-positive section expressed vimentin, snail, CD44, CD133 and NANOG. (**B**–**E**) Kaplan–Meier survival curve analyses of the association between survival probability and CXCR4, E-cadherin, CD133 and NANOG expression using the log rank test E-cadherin (**B**); CXCR4 (**C**), CD133 (**D**), NONOG (**E**) expression.

We first pathologically analyzed CXCR4 expression in 61 invasive ovarian cancer (OC) tissues (see Table 1). Specifically, the CXCR4 protein was expressed in 7/40 (17.50%) of paired benign ovarian tumor tissues but in 37/61 (60.66%) of tumor tissues, notably in the cytoplasm of tumor cells (Figure 1A). Additionally, CXCR4 expression was observed in tumor samples from patients; 68.90% of patients had lymph node metastasis, while 31.10% did not. CXCR4 expression was detected in 67.21% of cases with peritoneal cytology; the other 32.79% cases showed no nonperitoneal cytology. In addition, expression of CXCR4 was significantly related to poor histological grade and advanced FIGO stage (*P*=0.011 and *P*=0.019, respectively).

**Table 1:** Correlations between expression of CXCR4 and clinicopathological features.

**Characteristics**	**n (%)**	**CXCR4**		**χ2**	**P**
		**Positive**	**Negative**		
Age(yrs.)					
<47	27 (44.26)	14	13		
>47	34 (55.74)	23	11	1.573	0.322
FIGO stage					
I-II	18 (29.51)	15	3		
III-IV	43 (70.49)	22	21	5.503	0.019
Tumor grade					
I	26 (42.62)	11	15		
II-III	35 (57.38)	26	9	6.392	0.011
Lymphatic metastasis					
Positive	40 (65.57)	30	10		
Negative	21 (34.43)	7	14	10.018	0.002
Peritoneal cytology					
Positive	38 (62.30)	27	11		
Negative	23 (37.70)	10	13	4.565	0.033

### Analysis indicates a strong association of CXCR4 expression with EMT- and CSC-related proteins in epithelial ovarian cancer

In this study, we analyzed human epithelial ovarian cancer (EOC) tissue specimens. Among the EOC tissues examined, CXCR4 expression correlated strongly with expression of proteins related to the epithelial-mesenchymal transition (EMT) and cancer stem cell (CSC), including vimentin, N-cadherin, E-cadherin, CD44, CD133, and NANOG (Figure 1A and Supplementary Figure 1). Briefly, high CXCR4 expression was significantly associated with low levels of E-cadherin expression (*P*=0.001, r =0.405) but high levels of snail (*P*=0.135, r=0.193), vimentin (*P*<0.001, r=0.459), CD44 (*P*=0.229, r=0.135), CD133 (*P*<0.001, r=0.588) and NANOG (*P*=0.001, r=0. 412) expression (Table 2). Interestingly, not all benign ovarian tumor tissues were negative for CXCR4, with some weak expression observed. To evaluate this association of CXCR4 level with tumorigenesis, we further analyzed clinicopathological features and CXCR4, EMT- and CSC-related protein expression. Notably, aggressive clinicopathological features were more likely to be found in patients with high CXCR4 expression. For example, patients with high CXCR4 levels harbored more tumors with poor differentiation (Table 1; *P*=0.011) and expressed decreased levels of E-cadherin (*P*=0.001) and increased levels of vimentin, CD133, and NANOG proteins (Table 2; *P*<0.001). In addition, CXCR4 protein expression is lower in SKOV3 cell lines than the normal T80, which is associated with E-cadherin, vimentin, CD133, and NANOG, but not with snail and CD44 (Table 2 and Supplementary Figure 5). These could be due to two reasons: (i) cell lines may be changed during passage (cells can change in long term passage); (ii) patient samples may be from different subtypes of ovarian cancers.

**Table 2:** Association among CXCR4 and EMT-, stemness-related protein expressions in EOC tissue specimens.

**Variable**	**CXCR4, *n* (%)**			
	**Positive**	**Negative**	***r***	***P***
E-cadherin				
Positive	7 (11.48)	14 (22.95)		
Negative	30 (49.18)	10 (16.39)	-0.405	0.001
snail				
Positive	27 (44.27)	13 (21.31)		
Negative	10 (16.39)	11 (18.03)	0.193	0.135
vimentin				
Positive	28 (45.9)	7 (11.48)		
Negative	9 (14.75)	17 (27.87)	0.459	<0.001
CD44				
Positive	25 (40.98)	13 (21.31)		
Negative	12 (19.67)	11 (18.04)	0.135	0.229
CD133				
Positive	31(50.82)	8 (13.11)		
Negative	6 (9.84)	16 (26.23)	0.588	<0.001
NANOG				
Positive	29 (47.54)	9 (14.75)		
Negative	8 (13.12)	15 (24.59)	0.412	0.001

### Clinical prognostic examination shows a significant correlation of CXCR4, EMT- and CSC-related protein expression with survival rates in EOC patients

We followed up a total of 61 patients until March 2016, 38 of whom died over during the follow-up time. The 50-month survival rate was 37.7%. In our research, survival data were stratified depending on CXCR4 expression. Specifically, the EOC survival rates at 50 months were 29.73% (11/37) and 50.0% (12/24) for CXCR4^+^ and CXCR4^–^ tumors, 42.5% (17/40) and 28.57% (6/21) for E-cadherin^+^ and E-cadherin^–^ tumors, and 23.08% (9/39) and 63.64% (14/22) for CD133^+^ and CD133^–^ tumors, respectively. There were some excellent correlations between CXCR4, E-cadherin, CD133, and NANOG intensities and the median survival times (MSTs). MSTs were 11.0 m versus 24.0 m (*P*=0.018) for CXCR4+ compared to CXCR4^–^ tumors, 39.0 m versus 18.0 m (*P*=0.020) for E-cadherin^+^ compared to E-cadherin^–^ tumors, 17.0 m versus 26.0 m (*P*=0.019) for CD133+ compared to CD133^–^ tumors, and 16.0 m versus 32.0 m (*P*=0.028) for NANOG^+^ compared to NANOG^–^ tumors, respectively (Figure 1B–1E). Thus, the MSTs of CXCR4^+^, E-cadherin^–^, CD133^+,^ and NANOG^+^ were reduced in EOC patients (*P*<0.05). These results indicate that expression levels of CXCR4, E-cadherin, CD133 and NANOG are valuable predictors of survival in patients with EOC.

### CXCR4 knockdown diminishes the growth, colony forming ability, invasion capacity and epithelial–mesenchymal transition (EMT) of ovarian cancer cells *in vitro*

**Figure 2:**
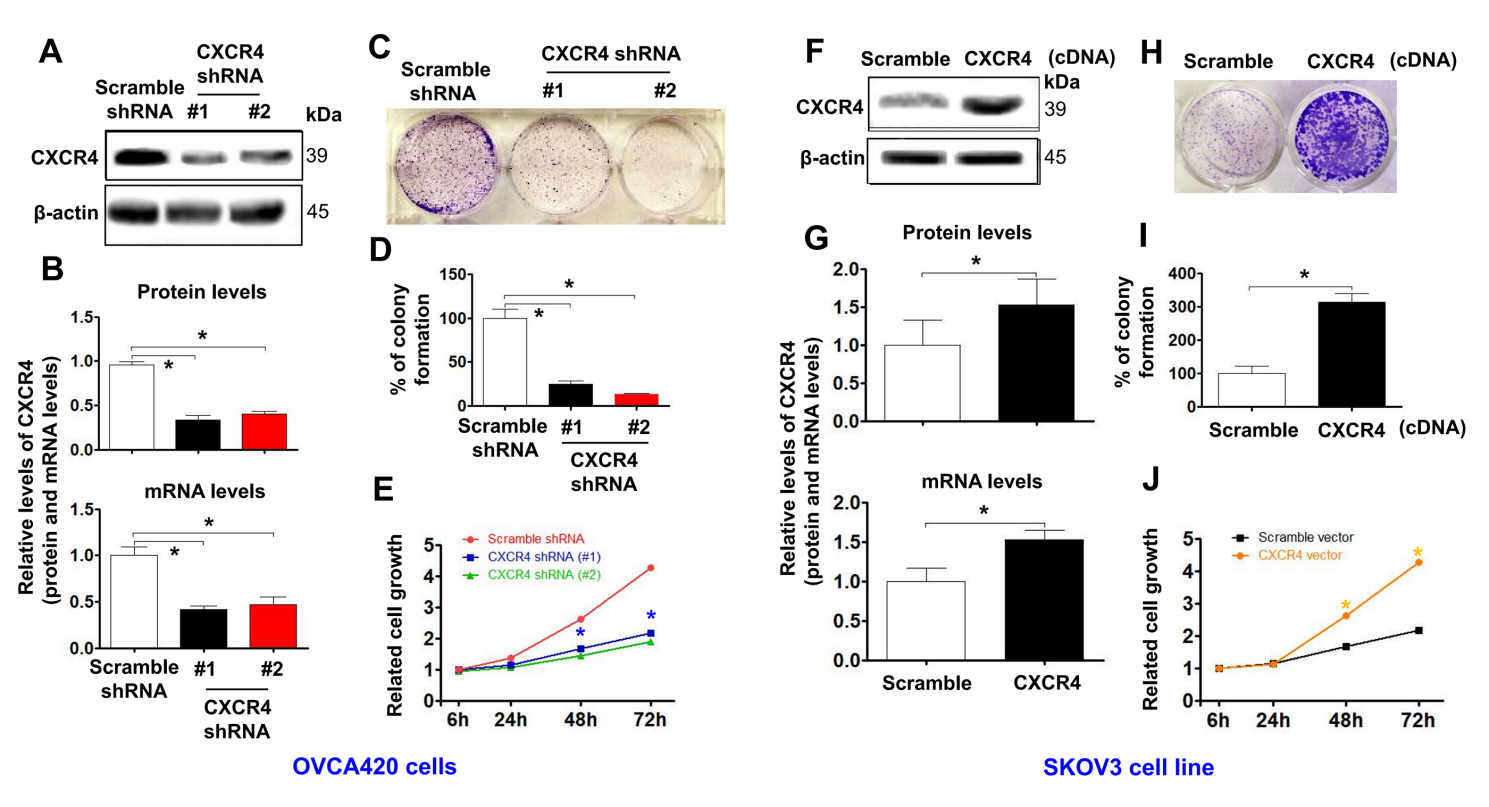
**Determining effects of CXCR4 knockdown on diminishing the cancer (EOC) proliferation capacity.** CXCR4 expression in CXCR4-kD#1/OVCA420 (CXCR4 shRNA#1), CXCR4-kD#2/OVCA420 (CXCR4 shRNA#2) (**A**, **B**) and CXCR4/SKOV3 (CXCR4 cDNA) (**F**, **G**) cells were analysed by Western blotting (WB) and qRT-PCR, respectively. The cancer (EOC) proliferation capacity was determined by colony formation assay (**C**, **H**) with percentage of colony formation (**D**, **I**) in both OVCA420 and SKOV3 stable cell lines modified by CXCR4 knockdown or overexpression, respectively. The MTT assay was used to measure the proliferation of the CXCR4-shRNA knockdown OVCA420 (**E**) and CXCR4-overexpressed SKOV3 (**J**), respectively. Absorbance was measured at 490 nm using the average from triplicate wells. Data are presented as the mean ± SD of three independent experiments. Asterisk indicates *P*<0.05 compared with the controls as determined by *t* test.

To evaluate the effects of CXCR4 expression on tumorigenesis in EOC, we examined CXCR4 expression in an epithelial cell line derived from the human ovarian surface and eight ovarian cancer cell lines. We discovered that the expression levels of CXCR4 mRNA and protein were very high in OVCAR420 cells and lowest in SKOV3 cells [Supplementary Figure 2 (upper panel); *P< 0.05]. Therefore, OVCAR420 cells were stably transfected with CXCR4 shRNAs or a negative control plasmid. As shown in Figure 2A, 2B, transfecting CXCR4shRNA#1 and CXCR4shRNA#2 remarkably decreased CXCR4 protein expression in OVCAR420 cells compared to scramble shRNA. Furthermore, qRT-PCR data showed that CXCR4 mRNA expression in OVCAR420 cells was reduced by both CXCR4 shRNA#1 and #2 knockdown compared with Scramble shRNA (Figure 2B, lower panel; *P<0.05). SKOV3 cells were stably transfected with the CXCR4 clone or control plasmid, and CXCR4 mRNA and protein expression levels were remarkably increased in CXCR4 cDNA-transfected SKOV3 cells (CXCR4 cDNA) (Figure 2F, 2G; *P< 0.05).

Additionally, CXCR4 knockdown using both CXCR4 shRNA#1 and #2 meaningfully reduced the colony forming ability (Figure 2C, 2D; *P<0.05) and tumorigenic growth (Figure 2E) of OVCAR420 cells compared to Scramble shRNA. Most importantly, CXCR4 overexpression significantly increased colony formation (Figure 2H, 2I; *P<0.05) and proliferation (Figure 2J; *P<0.05) of SKOV3 cells compared with Scramble cDNA transfection.

**Figure 3:**
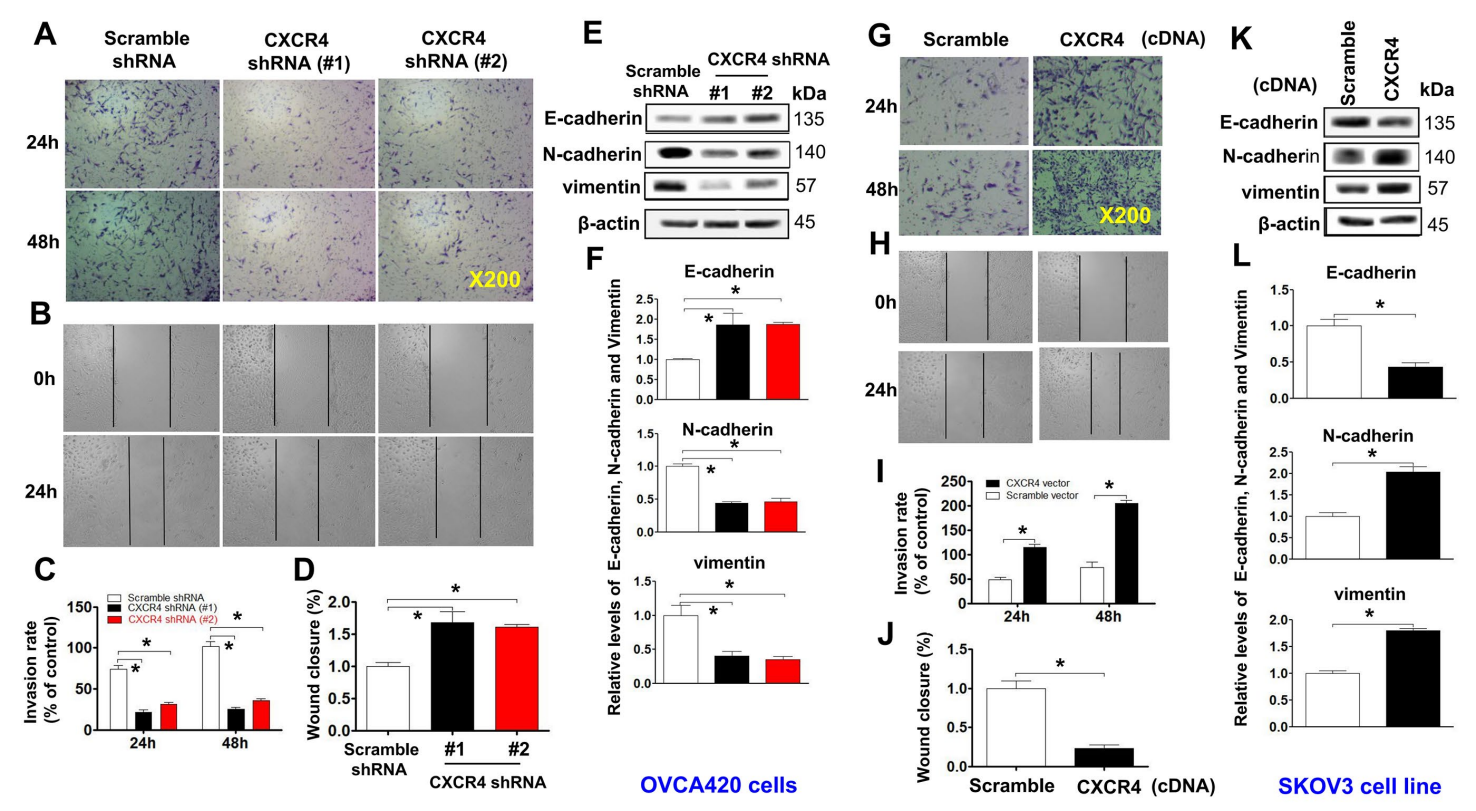
**Examining effects of CXCR4 knockdown on decreasing the cancer (EOC) invasion capacity.** A transwell tumour cell invasion assay showed that knockdown of CXCR4 reduced the invasion ability of OVCA420 cell lines (**A**) and that overexpression of CXCR4 enhanced the invasion ability of SKOV3 cells (**G**). The number of invaded cells were quantified by counting the total number of cells from 10 random fields (magnification, 200X) (**C**, **I**). A wound-healing assay showed that knockdown of CXCR4 reduced the migration ability of OVCA420 cells (**B**, **D**) and that overexpression of CXCRC4 enhanced the migration ability of SKOV3 cells (**H**, **J**), respectively. The effects of CXCR4 on the expression of EMT-related E-cadherin, N-cadherin and vimentin protein levels indicated in both CXCR4-knockdown OVCA420 (**E**) and -overexpressed SKOV3 (**K**) cell lines were analysed by WB with the indicated antibody against each protein examined, respectively. Band density ratios of each protein indicated to β-actin were determined by densitometry analysis (**F**, **L**). Data are presented as the mean ± SD of three independent experiments. Asterisk indicates P<0.05 compared with the controls as determined by t test.

Moreover, CXCR4 knockdown remarkably reduced the invasion capacity (Figure 3A, 3C;*P<0.05) and migration (Figure 3B, 3D; *P<0.05) of OVCAR420 cells compared to the scramble shRNA control. However, CXCR4 overexpression notably resulted in increased numbers of invading (Figure 3G, 3I; *P<0.05) and migrating (Figure 3H, 3J; *P<0.05) SKOV3 cells compared to scramble cDNA. The phenotypic/genotypic characteristics of stable ovarian cancer cells, such as EMT and stemness properties, were altered after CXCR4 overexpression or knockdown [25]. We used the typical EMT markers E-cadherin, vimentin, N-cadherin, and snail for comparisons among cells. Knockdown of CXCR4 expression significantly increased expression of E-cadherin but decreased that of N-cadherin, snail, vimentin, and slug in CXCR4 shRNA**-**transfected OVCAR420 cells (Figure 3E, 3F and Supplementary Figure 3A; *P<0.05). In contrast, overexpressing CXCR4 induced decreased E-cadherin expression but increased N-cadherin, vimentin, snail, and slug expression in SKOV3 cells (Figure 3K, 3L and Supplementary Figure 3B; *P<0.05). These data strongly suggest that CXCR4 plays a critical role in enhancing tumorigenesis of EOC.

### CXCR4 overexpression facilitates sphere formation and CSC stemness of ovarian cancer cells, which was markedly counteracted by CXCR4 knockdown

**Figure 4:**
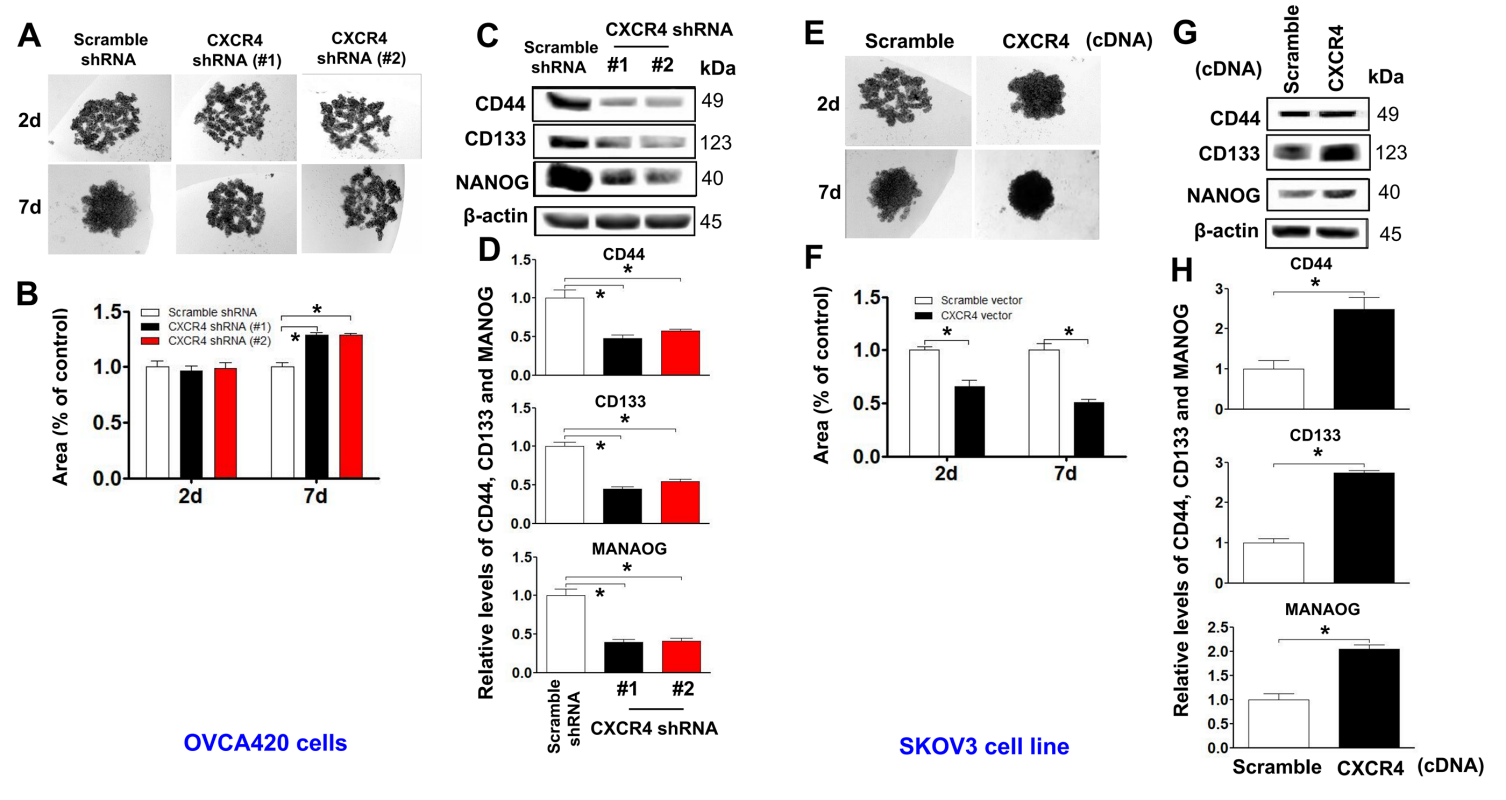
**Determining effects of CXCR4 overexpression on augmenting the cancer (EOC) spheroid formation capacity.** A spheroid culture in hanging drops assay showing that knockdown of CXCR4 reduced the spheroid formation ability of OVCA420 cells (**A**) and that overexpression of CXCRC4 enhanced the spheroid formation ability of SKOV3 cells (**E**), which were quantified by counting the total spheroid hanging drop area (percentage of control) from both OVCA420 and SKOV3 spheroid culture experiments, respectively (**B**, **F**). Accordingly, the CXCR4 effects on expression of CSC-related CD44, CD133 and NANOG proteins in both CXCR4-knockdowned OVCA420 and overexpressed SKOV3 cells were analysed by WB with the indicated antibody against each protein examined, respectively (**C**, **G**). Band density ratios of each protein indicated to β-actin were determined by densitometry analysis (**D**, **H**). Data are presented as the mean ± SD of three independent experiments. Asterisk indicates *P*<0.05 compared with the controls as determined by *t* test.

Stable cells with CXCR4 overexpression or knockdown (OVCAR420 and SKOV3) were gradually dissociated and seeded into hanging drops using serum-free medium (Figure 4A, 4E). CXCR4 knockdown-OVCAR420 cells appeared less tightly attached, and there were no clustered or overlapping cells in the three-dimensional (3D) configuration up to one week compared to scramble shRNA transfected-OVCAR420 cells (Figure 4B; *P<0.05). Notably, during the first 2 days, the Scramble shRNA-transfected cells appeared to be aggregated, merged and differentiated into 3D balls with a spheroid configuration in a nonadhesive and suspended environment. After culturing for 6-7 days, the spheres displayed a round and smooth shape (Figure 4A, left panel). During the first 2 days in the nonadhesive and suspended environment, most of the suspended SKOV3 cells overexpressing either CXCR4 or Scramble cDNA appeared to be aggregated, merged and differentiated into 3D balls in a spheroid configuration (Figure 4E, upper panels); the morphology of SKOV3 cells overexpressing CXCR4 then consisted of floating spheres. After culturing for 5 - 7 days, we observed more spheres with a rounder and smoother shape than the floating spheres produced by Scramble overexpression. These spheres gradually grew over time (Figure 4F; *P<0.05). Morphologically, the CXCR4-knockdown spheres appeared more tightly attached with more clustering or overlapping in the 3D configuration compared with the scramble cDNA-transfected SKOV3 spheres. At the same time, stable ovarian cancer cells with stem-like characteristics were altered after CXCR4 overexpression or knockdown [25]. Expression of the representative CSC markers CD44, CD133, NANOG, SOX2, and Oct-4 was compared between these cells: CXCR4 knockdown decreased CD44, CD133, NANOG, SOX2, and Oct-4 expression in OVCAR420 cells (Figure 4C, 4D and Supplementary Figure 4A; *P<0.05) whereas, CXCR4 overexpression increased CD44, CD133, NANOG, SOX2 and Oct-4 expression in SKOV3 cells (Figure 4G, 4H and Supplementary Figure 4B; *P<0.05).

### Inhibition of the PI3K/Akt/mTOR signaling pathway limits CXCR4 overexpression-mediated tumorigenic effects on EOC, EMT and CSC stemness *in vitro*, with significant increases by CXCR4 knockdown

**Figure 5:**
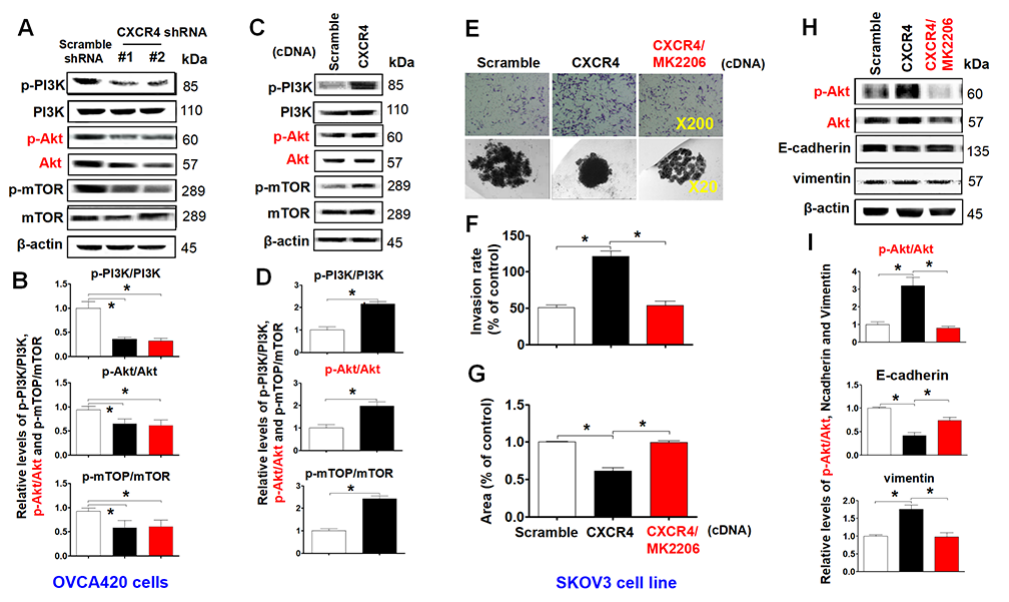
**Characterizing the role of the PI3K/Akt/mTOR pathway in promoting CXCR4 overexpression-mediated ovarian cancer invasion, EMT, and CSC stemness.** PI3K/Akt/mTOR pathway-related protein phosphorylated states indicated were analysed in both CXCR4 shRNA knockdowned-OVCA420 and CXCR4 overexpressed SKOV3 cells by WB with the indicated antibody against each protein, respectively (**A**, **C**). Band density ratios of phosphorylated-PI3K (p-PI3K), -Akt (p-Akt) and -mTOR to total-PI3K (PI3K), -Akt (Akt) and -mTOR (mTOR) were determined by densitometry analysis, respectively (**B**, **D**). Effects of MK-2206 on inhibiting SKOV3 cell invasion induced by CXCR4 overexpressing were analysed by a transwell tumour cell invasion assay (**E**, upper panel). Effects of MK-2206 on inhibiting SKOV3 cell spheroid formation capacity induced by CXCR4 overexpressing were analysed by a spheroid culture in hanging drops assay (**E**, lower panel), which were quantified by counting the total number of cells (invasion rate) from 10 random fields (magnification, 200X) (**F**), and the total spheroid hanging drop area (percentage of control) from the CXCR4-overexpressed SKOV3 culture cell experiments, respectively (**G**). Furthermore, effects of MK-2206 on inhibiting the expression of p-Akt, Akt, EMT-related proteins (E-cadherin, N-cadherin, vimentin and snail) in the CXCR4 overexpressed SKOV3 cells were analysed by WB with the indicated antibody against each protein examined (**H**). Band density ratios of p-Akt to Akt, E-cadherin, N-cadherin and vimentin to β-actin were determined by densitometry analysis, respectively (**I**). Data are presented as the mean ± SD of three independent experiments. Asterisk indicates *P*<0.05 compared with the controls as determined by *t* test. Please note that CSC related protein expression profiles in the CXCR4 overexpressed SKOV3 cell line in the presence or absence of MK-2206 treatment were described in the supplementary materials (Supplementary Figure 5).

To investigate the role of the PI3K/Akt/mTOR signaling pathway in mediating CXCR4 pathological functions, we examined phosphorylation of PI3K, Akt and mTOR in CXCR4-knockdown OVCA420 cells. Interestingly, both shRNA#1- and #2-mediated CXCR4 knockdown notably decreased phosphorylation of PI3K, Akt and mTOR and reduced PI3K/Akt/mTOR signaling activation in OVCA420 cells (Figure 5A, 5B; *P<0.05). In addition, we sought to determine whether CXCR4 overexpression alters the PI3K/Akt/mTOR signaling pathway in SKOV3 cells. Importantly, overexpressing CXCR4 markedly increased PI3K, Akt and mTOR phosphorylation in SKOV3 cells (Figure 5C, 5D; *P<0.05).

As MK2206 specifically targets the PI3K/Akt/mTOR signaling pathway, we applied this inhibitor and examined CXCR4-mediated EMT and CSC stemness and its related signaling pathway in SKOV3 cells. We found that MK2206 treatment markedly limited tumor cell invasion (Figure 5E, upper panel, Figure 5F;*P<0.05) and spheroid formation capacity (Figure 5E, lower panel, Figure 5G). Furthermore, in CXCR4-overexpressing SKOV3 cells, MK2206 significantly inhibited Akt phosphorylation and reduced CXCR4-mediated vimentin, CD44, CD133, NANOG, SOX2, Oct-4, N-cadherin, snail and slug protein expression (Figure 5H, 5I and Supplementary Figure 5A–5C; *P<0.05). Most interestingly, MK2206 recovered the E-cadherin protein expression reduced by overexpressing CXCR4 in SKOV3 cells (Figure 5H, 5I; *P<0.05). These data clearly suggest that blocking the PI3K/Akt/mTOR pathway is a potential target for CXCR4-associated ovarian cancers.

### Characterization of the role of CXCR4 in reducing and advancing the sensitivity of EOC cells to paclitaxel *in vitro*

To clarify the mechanism by which CXCR4 modulates the proliferation rate of ovarian cancer cells, we next analyzed the viability of stable CXCR4-shRNA#1, #2-mediated knockdown-OVCAR420 (CXCR4-Kd#1/OVCAR420; CXCR4-Kd#2/OVCAR420) and CXCR4-overexpressing SKOV3 (CXCR4/SKOV3) cell lines after treatment with paclitaxel (PTX) at 0.01 to 1000 nM for 48 hours by the MTT assay. In comparison with control cells, MTT assay results indicated that the IC50 of PTX in CXCR4-Kd#1/OVCAR420, CXCR4-Kd#2/OVCAR420 and Scramble-Kd/OVCAR420 cell lines was 5.77 nM, 3.6 nM and 28.14 nM, respectively. Thus, that the sensitivity of CXCR4-Kd#1/OVCAR420 and CXCR4-Kd#2/OVCAR420 cell lines to PTX was significantly increased by silencing CXCR4 expression compared to the control (Supplementary Figure 6A;*P< 0.05). These data indicate that knockdown of CXCR4 expression in the OVCAR420 cell line results in increased sensitivity to PTX treatment. In contrast, the IC50 of PTX in the CXCR4/SKOV3 and Scramble/SKOV3 cell lines was 189.3 nM and 35.12 nM, respectively, which indicated that the sensitivity of the CXCR4/SKOV3 cell line to PTX was significantly decreased due to overexpression of CXCR4 compared to the control (Supplementary Figure 6B; *P< 0.05). These data suggest that reducing or silencing expression of CXCR4 might result in increased sensitivity to PTX treatment in CXCR4-positive cell lines.

### CXCR4 knockdown synergistically sensitizes PTX chemotherapy antitumor effects in a xenograft tumor nude mouse model

**Figure 6:**
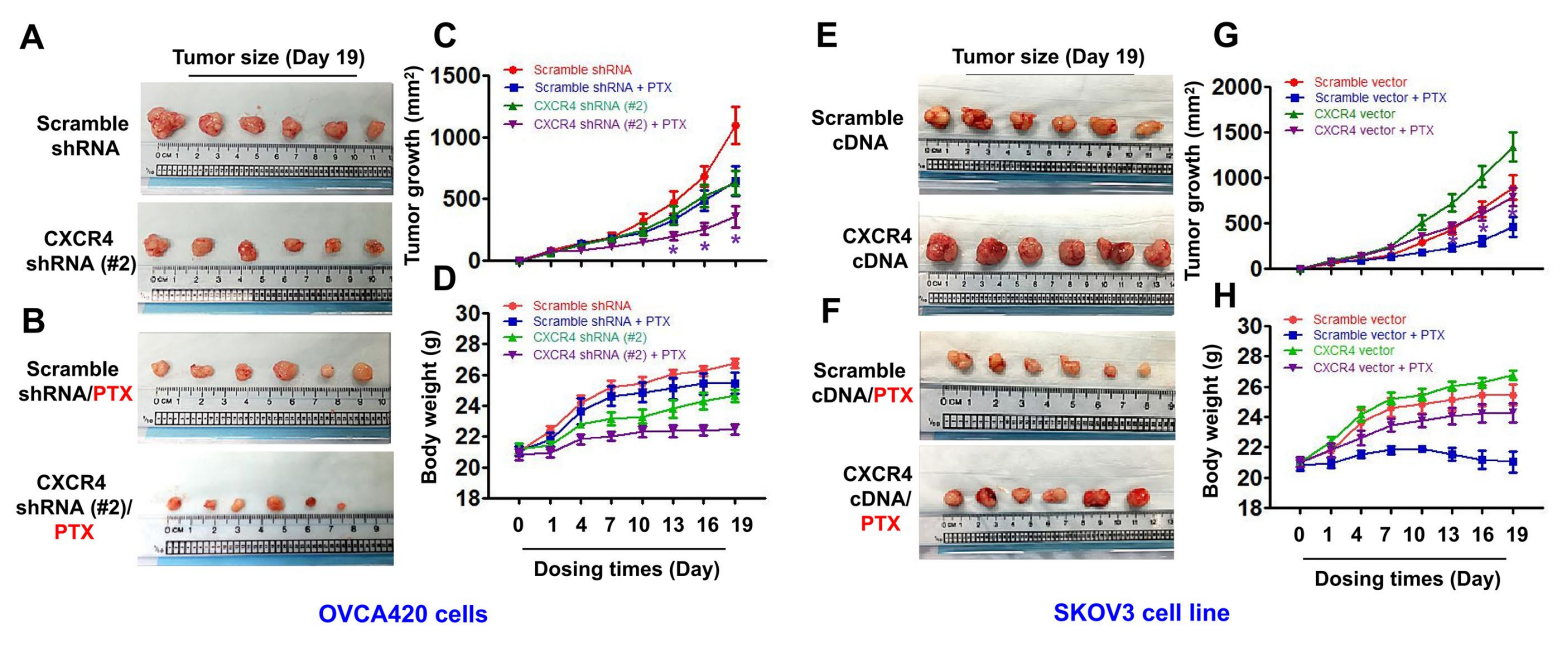
***In vivo* evaluating the effects of CXCR4 knockdown and overexpression chemosensitivity of both OVCA420 and SKOV3 tumour cells to PTX treatment in the xenograft tumour nude mouse model.** Representative images (day 19) were recorded under macro view representing the size of tumours in the tumour xenograft mice (**A**, **E**), when combined with PTX treatment (**B**, **F**). Tumour volume (**C**, **G**) and body weight (**D**, **H**) changes following CXCR4 knockdown and overexpression and, the combined treatment with paclitaxel (PTX) in the OVCA420 and SKOV3 tumour cell xenograft model, respectively. Data represent the means ±S.D. from six nude model mice for each time point examined. Asterisk indicates *P*< 0.05 compared with the controls as determined by *t* test.

To determine whether CXCR4 expression plays an important role in limiting PTX chemotherapy sensitivity *in vivo*, we developed CXCR4-Kd#2/OVCA420 and CXCR4/SKOV3 cell line-xenograft tumor nude mouse models. When the average diameter of the tumors reached 6 - 7 mm, the mice were i.p. treated with PTX (20 mg/kg) once every three days until day 19, as indicated. As shown in Figure 6A–6C, the mean tumor volume of mice in the CXCR4-Kd#2/OVCA420 group (lower panel) was reduced at days 15 and 19 compared with the Scramble-Kd/OVCA420 group (upper panel) (*P<0.05). Most importantly, CXCR4 knockdown synergistically enhanced the effect of PTX on inhibiting tumor growth (Figure 6A–6C, lower panel;*P<0.05). The tumor volume of mice in the CXCR4-Kd#2/OVCA420 group treated with PTX (Figure 6B, lower panel) was markedly reduced at days 13, 16 and 19 compared with mice in the Scramble-Kd/OVCA420 group treated with PTX (Figure 6B, upper panel). Conversely, as expected, the mean tumor volume of mice in the CXCR4/SKOV3 group (Figure 6E, lower panel) was significantly increased at days 10, 13, 15 and 19 compared with the Scramble/SKOV3 group (Figure 6E, upper panel). Strikingly, CXCR4 overexpression markedly abolished the effect of PTX on reducing tumor size (Figure 6F, 6G; *P<0.05). However, the tumor volume of mice in the CXCR4/SKOV3 groups treated with PTX (Figure 6F, lower panel) did not change compared with mice in the Scramble/SKOV3 group (Figure 6E, upper panel). These data are consistent with the results for mouse body weight measurements (Figure 6D, 6H).

### CXCR4 knockdown enhances the sensitivity of xenograft tumor cells to PTX treatment in a xenograft nude mouse model by decreasing the PI3K/Akt/mTOR signaling pathway and CXCR4-mediated EMT- and CSC-related protein expression

**Figure 7:**
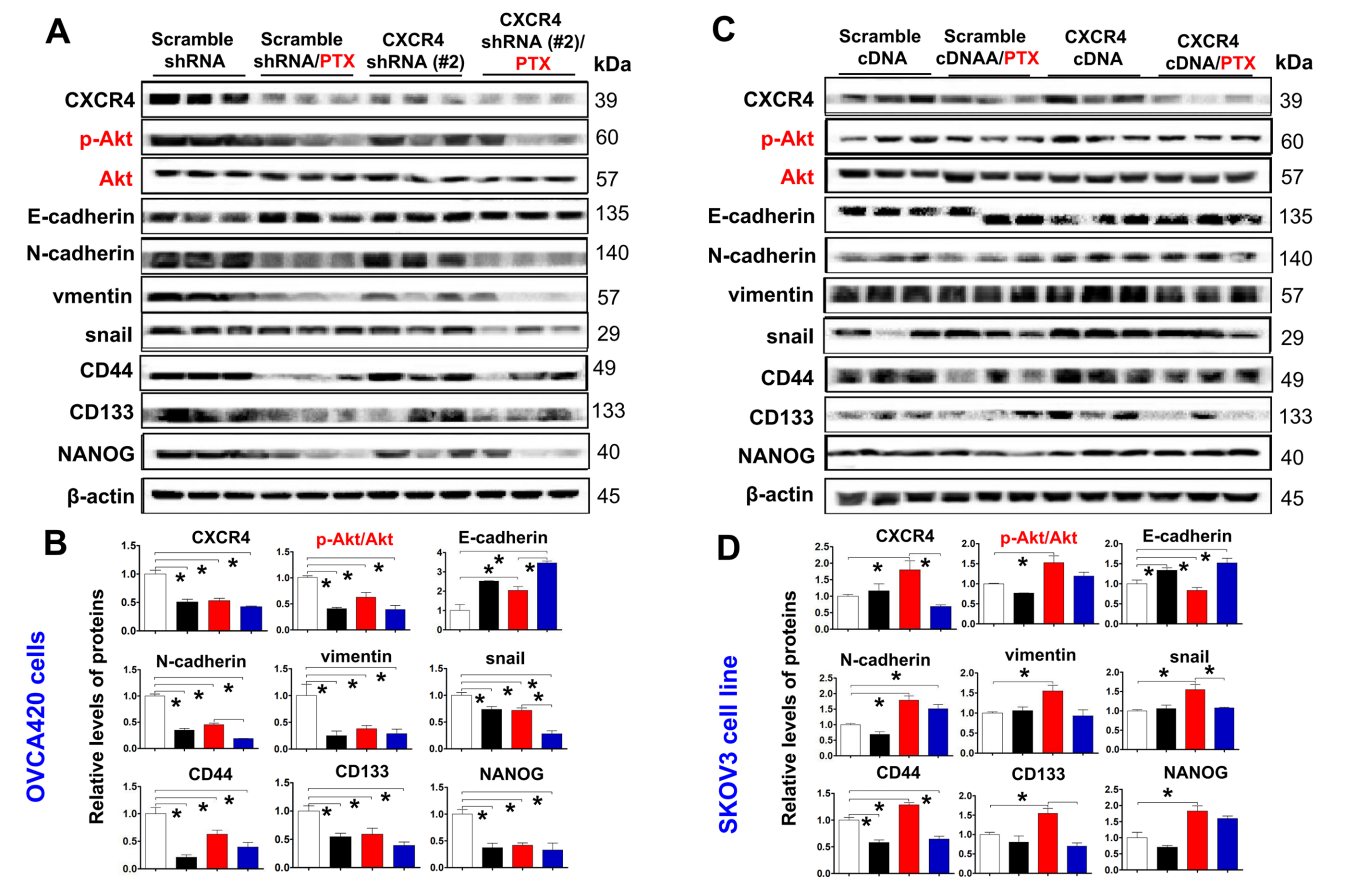
***Ex vivo* evaluating the role of CXCR4 knockdown in improving PTX chemosensitivity through reduction of PI3K/Akt/mTOR signalling, EMT- and CSC-related protein expressions.** CXCR4, EMT-and CSC-related protein expressions, as well as PI3K/Akt/mTOR signalling pathway in both OVCA420 (**A**) and SKOV3 (**C**) cells derived from the tumour xenograft model were analysed by WB using the indicated antibody against each protein examined. Band density ratios of p-Akt to Akt, and CXCR4, E-cadherin, N-cadherin, vimentin and snail, CD44, CD133 and NANOG to β-actin were determined by densitometry analysis, respectively (**B**, **D**). Data are presented as the mean ± SD of three independent experiments with triplicated wells for each condition. Asterisk indicates *P*< 0.05 compared with the control as determined by *t* test.

**Figure 8:**
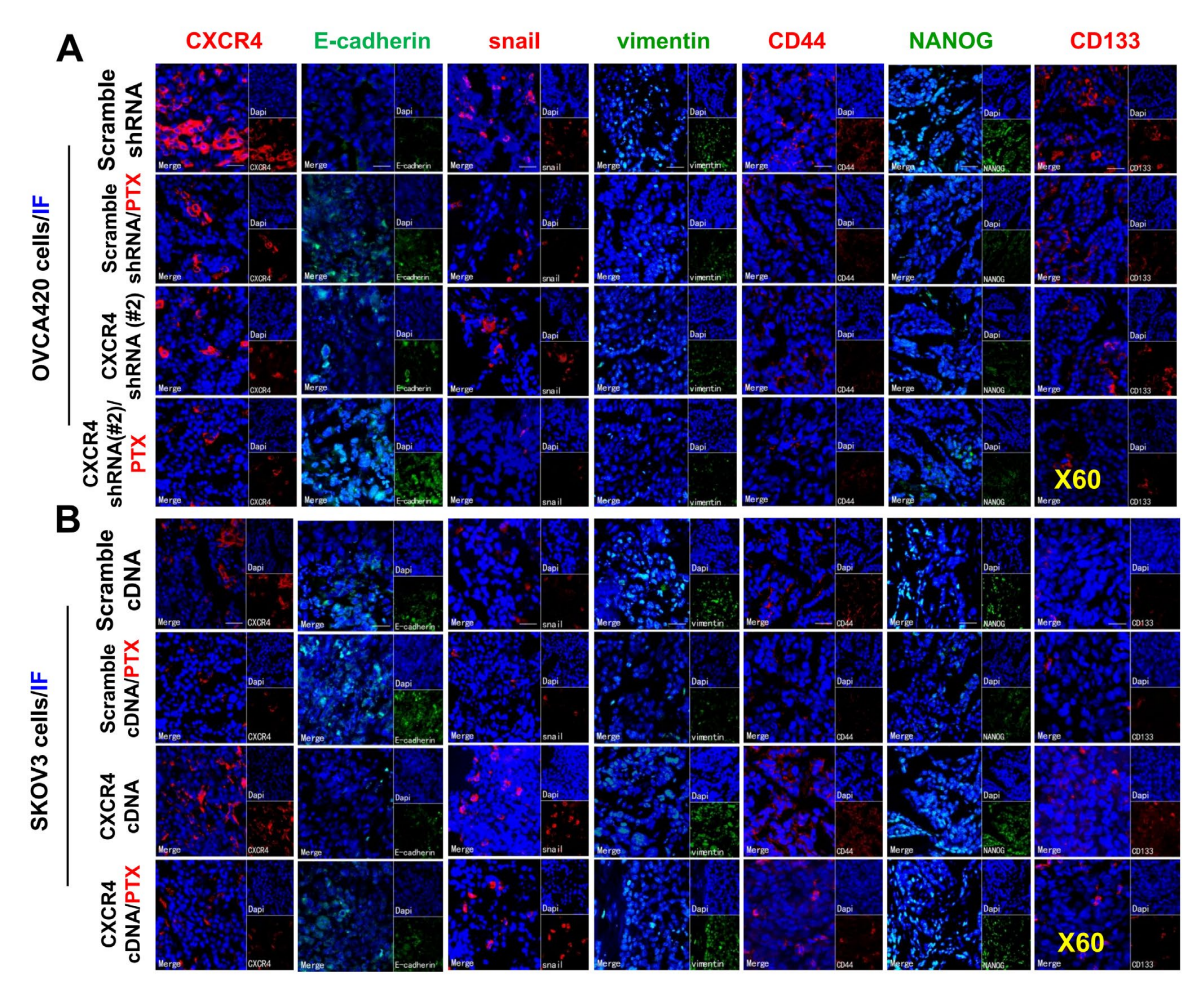
**Further *ex vivo* IF examining the expression of CXCR4, EMT- and CSC-related proteins in the OVCA420 and SKOV3 cell xenograft tissues from the tumours nude mice following treatment with PTX.** Immunofluorescent (IF) staining analysis images illustrated the location of CXCR4, EMT-, and CSC-related protein expressions in the OVCA420 (**A**) and SKOV3 (**B**) cell xenograft tumour tissues. Notably, red fluorescence shows the membrane expression of CXCR4, snail, CD44, and CD133; green fluorescence shows the membrane expression of E-cadherin, vimentin, and NANOG; and blue fluorescence shows all cell nuclei stained with DAPI (4′, 6-diamidino-2-phenylindole). Data represent one of the three independent experiments with similar results. Scale bars=50 μm.

To further confirm the effect of the PI3K/Akt/mTOR signaling pathway in CXCR4-induced EMT and CSC stemness *in vivo*, we used WB and immunofluorescence (IF) to investigate CXCR4-, EMT- and CSC-related protein expression, specifically the Akt signaling pathway, in CXCR4-kD#2/OVCA420 and CXCR4/SKOV3 tumor tissues derived from xenograft tumor nude mice treated with PTX or control. Clearly, both WB and IF showed that shRNA silencing CXCR4 synergistically enhanced the effect of PTX treatment on decreasing Akt phosphorylation and the resulting reduction in N-cadherin, vimentin, snail, CD44, CD133, and NANOG protein expression in OVCA420 tumor tissues (Figure 7A, 7B, Figure 8A). Specifically, silencing CXCR4, especially when combined with PTX treatment, notably increased E-cadherin protein expression (Figures 7A, 7B, 8A and Supplementary Figure 7A; *P<0.05). Conversely, CXCR4 overexpression strikingly counteracted the effect of PTX on decreasing Akt phosphorylation and reductions in N-cadherin, vimentin, snail, CD44, CD133, and NANOG protein expression in SKOV3 tumor tissues (Figures 7C, 7D, 8B and Supplementary Figure 7B; *P<0.05). Most interestingly, PTX treatment markedly abolished the tumorigenic effect of CXCR4 overexpression on decreasing E-cadherin expression (Figure 7C, 7D; *P<0.05).

## DISCUSSION

EOC is characterized by high proliferation and invasion, metastasis in the early stage, the formation of CSCs, chemotherapy resistance and recurrence compared to other malignant diseases. To develop new prognostic markers of disease and therapeutic targets of treatment in EOC, significant efforts have been made to clarify the molecular mechanisms underlying the invasion and metastasis of EOC over the past several decades. Nevertheless, the detailed mechanisms of EOC metastasis remain unknown. Among chemokine signaling axes, the critical role that the SDF-1/CXCR4 axis plays in various solid tumors has been demonstrated [26]. CXCR4 was originally identified as a cofactor for HIV; it is a G protein-coupled transmembrane receptor that enters CD4^+^ T cells [27]. CXCR4 activation with SDF-1 triggers G protein signaling, which stimulates a number of intracellular signal transduction pathways and molecules that regulate migration, adhesion, cell survival, chemotaxis, and proliferation [28–30]. However, there is scarce research on the functional roles of CXCR4 in the EMT and CSC features of EOC, and the molecular pathways by which CXCR4 increases EMT and CSC stemness deserve further study.

Recently, a novel perspective for determining ovarian cancer biological behavior has been promoted by the cancer stem-cell theory [31, 32], highlighting the concept of CSCs and their newly identified roles in tumor biology involving the CXCL12–CXCR4 axis. CXCR4 expression/overexpression has been detected in various cancer histotypes, including lung [33], pancreatic [34], colon [35], breast [36], renal [37] and prostate histotypes [38], which are derived from CSCs. A previous study reported that the CXCR4 pathway influences EMT in *in vitro* models comprising pancreatic cancer cells [39], and increasing evidence has revealed that tumor metastasis is related to EMT [40]. The EMT process also involves stemness maintenance and differentiation of cells into numerous cell types in the initiation and development of metastasis [41]. The concept of EMT is integrated with CSCs in cancer biology [42]. Indeed, experimental data show that the EMT process induces CSC features in many epithelial cancer cell lines [43, 44]. According to these studies, induction of EMT sustains CSC features. Thus, CSC properties are relevant to EMT induction. Thus, EMT induction and CSC properties should strongly affect EOC pathogenesis. In our research, we elaborated on the clinical significance of CXCR4-, EMT- and CSC-related genes in EOC and the mechanistic role that CXCR4 plays in regulating EOC metastasis. The findings suggest that altered CXCR4 expression is linked to changes in ovarian CSC stemness. Functionally, overexpression of CXCR4 induced EMT, stem-like behavior and motility of EOC cells *in vitro* and accelerated tumor xenograft growth *in vivo*. In contrast, CXCR4 knockdown countered these effects not only *in vitro* but also *in vivo*.

Nevertheless, it remains unknown whether CXCR4-mediated signaling in ovarian cancer is involved in PI3K/Akt/mTOR pathway regulation and impacts EMT and CSC characteristics, invasion and metastasis. Ovarian cancer cells that acquire stem-like invasiveness through the combined PI3K/Akt pathway undergo EMT [45], and increasing evidence reveals that EOC metastasis correlates with EMT [46–48]. Our study shows that an inhibitor of the PI3K/Akt/mTOR pathway, MK-2206, suppressed PI3K/Akt/mTOR pathway activation and abolished the EMT, CSC stemness, and invasion induced by CXCR4, most likely because the PI3K/Akt/mTOR pathway plays an important role in tumor cell EMT behavior and CSC stemness regulation in EOC cells. However, a recent study reported that the Akt inhibitors NVP-BEZ-235 and NVP-LDE-225 function cooperatively to suppress EMT in pancreatic CSCs and that the PI3K/Akt/mTOR pathway is aberrantly reactivated in pancreatic CSCs [49]. These data indicate that CXCR4 may serve as a molecular marker for the treatment and disease prognosis of EOC. Regardless, further research on whether EMT can be activated by CXCR4 through other mechanisms or whether PI3K/Akt/mTOR-induced EMT and CSC characteristics are related to other transcription factors needs to be performed.

**Figure 9:**
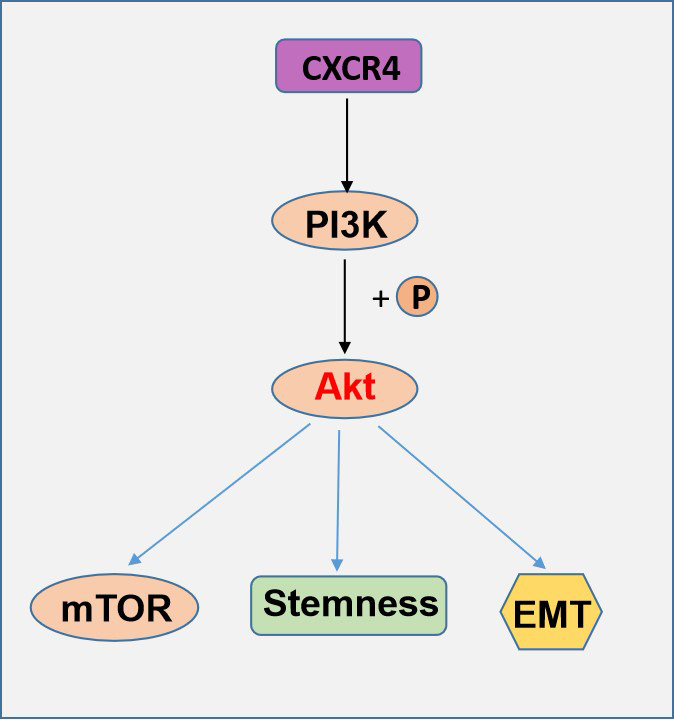
A schematic model of CXCR4 contributes distinctively to PI3K/Akt/mTOR pathway effects in EOC.

In summary, our study clarified that CXCR4 contributes distinctively to PI3K/Akt/mTOR pathway effects in EOC. Additionally, chemotherapy resistance is enhanced by the increased formation of CSCs and EMT by the CXCR4-PI3K/Akt/mTOR axis. The exact role of CXCR4 in sphere formation and acquisition of stem cell characteristics has remained unknown to date, but this study describes for the first time the significant role of CXCR4 in ovarian CSCs. All of the results from this study indicate that CXCR4 and PI3K/Akt/mTOR may act as a target for combination therapy to inhibit EOC chemotherapy resistance and metastasis (Figure 9).

## MATERIALS AND METHODS

### Patients and tissue samples

Our research was approved by the Ethics Committee of Clinical Research of The First Affiliated Hospital, Gui Zhou Medical University (Guiyang, China). All patients who underwent surgery for resection of EOC lesions at The First Affiliated Hospital, Gui Zhou Medical University signed informed consent forms. We obtained tissue specimens from 61 ovarian cancer patients and 40 benign epithelial ovarian tumor patients between January 2010 and January 2012. No patients had received chemotherapy or radiotherapy before the surgery. All cases were histologically diagnosed, as indicated in Table 1. We retrieved cancerous and benign ovarian tumor tissues from paraffin tissue blocks for retrospective analysis.

### Immunohistochemistry staining analysis

For tumor immunohistochemistry (IHC), formalin-fixed, paraffin-embedded tumor microarray slides were subjected to deparaffinization, hydration, epitope retrieval, and probing with a rabbit polyclonal antibody, as shown in Table 2. For the negative control, sections were treated with phosphate-buffered saline (PBS) without the primary antibody.

### Quantitative reverse transcriptase polymerase chain reaction (qRT-PCR)

We reverse-transcribed RNA isolated from cells. We designed real-time RT-PCR primers as follows: CXCR4 forward 5′-GATCAGCATCGACTCCTTCA-3′ and reverse 5′-GGCTCCAAGGAAAGCATAGA-3′, and GAPDH (internal control) forward: 5′-AACGGGAAGCTTGTCATCAATGGAAA-3′, reverse: 5′-GCATCAGCAGAGGGGGCAGAG-3′. Every measurement was carried out in triplicate and repeated two times. We used the 2–ΔΔCt method, a relative quantification approach, to normalize the mRNA expression level of CXCR4 to that of GAPDH.

### Cell lines and cell culture

We obtained Dulbecco’s modified Eagle’s medium from Dr Chengxiong Xu (Moffitt Cancer Center and Research Institute, Tampa, USA) for growth of EOC cell lines (OVCAR2008, OVCAR3, OVCAR4, OVCAR420, OVCAR433, OVCAR5, SKOV3, C13, A2780cp). T80 human ovarian surface epithelial cells obtained from American Type Culture Collection (ATCC; Manassas, VA, USA) were cultured in specific medium according to the manufacturer’s instructions. All media contained sodium bicarbonate, 10% heat-inactivated FBS and phenol red, L-glutamine, L-cysteine, and L-methionine. We cultured the cells in an incubator under 5% CO_2_/95% air humidified conditions at 37° C.

### Plasmid cDNA construction and gene transduction

Our research used four CXCR4 shRNAs to target different regions of CXCR4 [GenBank: NM_003467]. The CXCR4 shRNA information is documented in (Supplementary Table 1). We cloned the shRNA oligonucleotides in the lentiviral vector pLKO.1 following the instructions provided by GE Healthcare Dharmacon, Inc. (Lafayette, CO, USA). The CXCR4 shRNA pLKO.1 constructs were cotransfected with the psPAX2 and pMD2.G plasmids into HEK-293T packaging cells using Lipofectamine 2000 (Life Technologies). All of the constructs were checked by sequencing, and stable transfection was performed according to the instructions of Lipofectamine 2000 (Invitrogen, Carlsbad, CA, USA). The plasmid pLX304-Blast-V5-CXCR4 was also cotransfected with psPAX2 and pMD2.G plasmids into HEK-293T packaging cells using Lipofectamine 2000. The primers are provided in (Supplementary Table 1).

### Western blotting analysis

Cells were lysed, and proteins were isolated. A total of 30 μg of protein was examined by Western blotting (WB) using the antibodies shown in (Supplementary Table 2). After washing with 0.05% Tween-20/TBS, the membranes were cultured with HRP-conjugated secondary antibodies and developed using an ECL kit (Amersham Biote). All antibodies were acquired from Santa Cruz Biotechnology, Inc. (Santa Cruz, CA, USA). Each sample was processed for WB three times.

### Colony formation assay

Stably transformed cells were incubated at a density of 300/well in 6-well plates, which were cultured in complete medium at 5% CO2 and 37° C for 14 days; the growth medium was refreshed every three days. The cells were stained with crystal violet solution (Sigma-Aldrich Co., LLC. Saint Louis, MO, USA) and methanol for 10 min at room temperature, after which the cells were counted using Quantity One analysis software (BioRad, Inc.). A colony was considered to be a group of cells of more than 50.

### *In vitro* invasion assays

The insert membranes of Transwells (8.0 μm pore; Millipore) in 12-well plates were precoated with 15 μg of Matrigel (Becton Dickinson Bioscience, Bedford, MA, USA). Cells (4x10^4^) were added to the upper chamber in 400 μL of growth medium that did not contain FBS, and normal culture medium was added to the lower chamber. We used a swab to remove the cells on the topmost surface of the membrane; the invading cells that adhered to the underside of the membrane were stained with crystal violet solution. Cells in 10 random fields were counted under a microscope after 1 and 2 days of incubation.

### Cell proliferation assay

Cells were cultured in 96-well plates at 1,500 cells/well after twenty-four hours of transfection. We used the MTT assay (Sigma, USA) to examine relative cell growth each day. Ten microliters of 5 mg/ml MTT was added to the media and incubated for 4 hours at 37° C. After removing the culture medium, the crystals were dissolved with 100 μL DMSO (Sigma), and absorbance at 490 nm was measured.

### Wound-healing assay

Stable cells were counted and seeded in 6-well plates at a density of 10^6^ cells/well in triplicate. A 200 μL pipette tip was used to scratch the attached cells, and a microscope captured images at the time the cells reached 70-80% confluence. The plate was incubated at 37° C and 5% CO2 for 1 day, and images of the same wounds were taken. The wound width was analyzed with ImageJ software (version 1.44, NIH), and the percentage of wound closure was compared to that at time 0 (mean ± SD). We calculated the percentage of migration as the initial width of the same scratch multiplied by 100 and divided by the scratch width. Each scratch was analyzed in at least five fields, and each sample was performed in duplicate.

### Spheroid culture by the hanging drop assay

We prepared stable cells as single-cell suspensions and suspended them again in serum-free medium (DMEM) to a density of 1.0×10^6^ cells/mL in a sterile vessel after counting. A 6-channel pipette was used to prepare a culture dish with 6 rows of 20 μL drops (200 cells/drop) in the inner surface of an inverted tissue lid. Then, the lid was overturned and placed on top of a culture dish containing 10 mL PBS. The drops were cultured at 37° C and 10% CO_2_ for 7 days. After that, we added 2-5 μL of fresh growth medium to the hanging drops every other day to maintain a volume of 20 μL. A microscope was used to image the resulting spheroids.

### Cell viability assay

We used the MTT assay to investigate the effect of paclitaxel (PTX) on the viability of A2780cp and C13 cell lines and measured absorbance at 570 nm with a Synergy H4 Hybrid microplate reader (BioTek, Inc., Winooski, VT, USA). IC50 values were calculated based on the relative viability versus the concentration of PTX.

### Nude mouse xenograft assay

The Animal Experiment Administration Committee of the University of South Florida, Florida, USA (IACUC Protocol#511) approved the animal experiments. We purchased athymic nude mice (6-8 weeks, 20±5 g) from Harlan Laboratory, Inc. (Tampa, USA). We chose two stable cell lines (OVCAR420 and SKOV3) transfected with CXCR4 shRNA#1 and #2 and scramble shRNA (named CXCR4-Kd#1/OVCAR420, CXCR4-Kd#2/OVCAR420 and Scramble-Kd/OVCAR420, respectively) and with CXCR4 cDNA plasmid and Scramble cDNA plasmid (named CXCR4-SKOV3 and Scramble-SKOV3, respectively) for this experiment. Seven groups of mice (six mice per group) were used for each cell line. The mice were subcutaneously injected with approximately 1x10^7^ cells suspended in 200 μL of PBS. Tumor formation was measured and documented as the tumor was visible to the naked eye. The tumor volume was calculated based on the formula for length x width^2^ x 0.5 PTX (20 mg/kg) was intraperitoneally (i.p.) injected when the average diameter of the tumors reached 6-7 mm. Individual treatments were administered once every three days for six weeks. After treatment, all mice were sacrificed by carbon dioxide anesthesia, and the tumor weights were measured. We excised and weighed the tumors at thirty days after the initial inoculation and analyzed them by WB and immunofluorescence (IF) staining.

### Statistical analysis

The data are shown as the mean ± standard deviation (SD). Comparisons of multiple groups were conducted by one-way analysis of variance (ANOVA) after Tukey’s multiple comparison test. Statistical significance was considered at values of *P*<0.05. Assays were performed at least three times independently.

### Availability of data and materials

All data generated or analyzed during this study are included in this published article.

## Supplementary Material

Supplementary Figures

Supplementary Tables
