# Correction for: CXCR4 knockdown enhances sensitivity of paclitaxel via the PI3K/Akt/mTOR pathway in ovarian carcinoma

**DOI:** 10.18632/aging.204367

**Published:** 2022-11-15

**Authors:** Dan Zi, Qing Li, Cheng-xiong Xu, Zhi-Wei Zhou, Guan-Bin Song, Cheng-Bin Hu, Fang Wen, Han-Lin Yang, Lei Nie, Xing Zhao, Jun Tan, Shu-Feng Zhou, Zhi-Xu He

**Affiliations:** 1 Department of Obstetrics and Gynecology, Guizhou Provincial People’s Hospital, Guiyan, Guizhou, 550002, China; 2 Department of Obstetrics and Gynecology, Affiliated Hospital of Guizhou Medical University, Guiyang, 550004, China; 3 Key Laboratory of Adult Stem Cell Transformation Research, Chinese Academy of Medical Sciences/Stem Cell and Tissue Engineering Research Center, Guizhou Medical University, Guiyang, 550004, China; 4 Key Laboratory of Endemic and Ethnic Diseases and Key Laboratory of Molecular Biology, Ministry of Education, Guizhou Medical University, Guiyang, 550004, China; 5 Department of Pharmaceutical Sciences, College of Pharmacy, University of South Florida, Tampa, FL 33612, USA; 6 Cancer Center, Daping Hospital and Research Institute of Surgery, The Third Military Medical University, Yuzhon, Chongqing, 40042, China; 7 Department of Radiation Oncology, University of Texas Southwestern Medical Center, Dallas, TX 75390, USA; 8 Key Laboratory of Biorheological Science and Technology, Ministry of Education, College of Bioengineering, Chongqing University, Chongqing, 400030, China; 9 Department of Computer Science and Engineering, University of South Florida, Tampa, FL 33620, USA; 10 Department of Bioengineering and Biotechnology, College of Chemical Engineering, Huaqiao University, Xiame, Fujian, 361021, China; 11 Department of Pediatrics, Affiliated Hospital of Zunyi Medical University, Zunyi, 563000, China

**This article has been corrected:** The authors replaced the “CXCR4shRNA (#1)/24h” image of the OVCA420 tumor cells used in the transwell invasion assay illustrated in **Figure 3A.** That image partially overlapped the “CXCR4shRNA (#1)/48h” image due to accidental mislabeling. Replacement was done using a representative image from the “CXCR4shRNA (#1)/24h” group from the original set of experiments.

The authors also corrected **Figure 5E** (SKOV3 cell invasion induced by CXCR4 overexpression, upper panel), where the control “Scramble” group image of SKOV3 cells was a duplication of the “Scramble” image in **Figure 3G**. The authors reused the same image because the cells used in **Figures 3G** (48-hour SKOV3 Scramble group) and **5E** (48-hour SKOV3 Scramble group) were from the same batch of experiments. To avoid misinterpretation, the authors have replaced the “Scramble” image in **Figure 5E** (upper panel) with a representative image from the 48-hour SKOV3 Scramble group from the original set of experiments.

These alterations do not affect the results or conclusions drawn in this work.

Corrected **Figures 3** and **5** are presented below.

**Figure 3:**
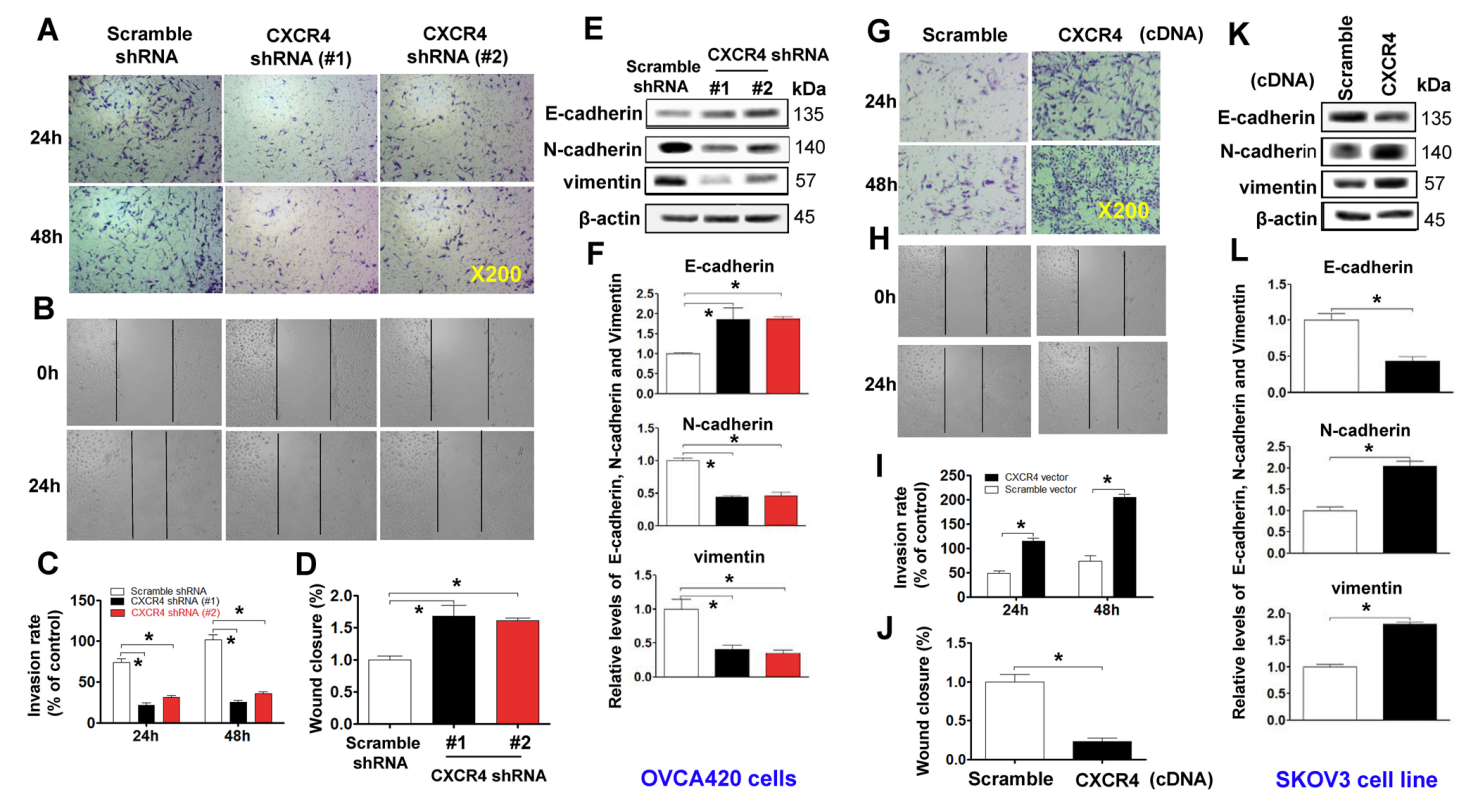
**Examining effects of CXCR4 knockdown on decreasing the cancer (EOC) invasion capacity.** A transwell tumour cell invasion assay showed that knockdown of CXCR4 reduced the invasion ability of OVCA420 cell lines (**A**) and that overexpression of CXCR4 enhanced the invasion ability of SKOV3 cells (**G**). The number of invaded cells were quantified by counting the total number of cells from 10 random fields (magnification, 200X) (**C**, **I**). A wound-healing assay showed that knockdown of CXCR4 reduced the migration ability of OVCA420 cells (**B**, **D**) and that overexpression of CXCRC4 enhanced the migration ability of SKOV3 cells (**H**, **J**), respectively. The effects of CXCR4 on the expression of EMT-related E-cadherin, N-cadherin and vimentin protein levels indicated in both CXCR4-knockdown OVCA420 (**E**) and -overexpressed SKOV3 (**K**) cell lines were analysed by WB with the indicated antibody against each protein examined, respectively. Band density ratios of each protein indicated to β-actin were determined by densitometry analysis (**F**, **L**). Data are presented as the mean ± SD of three independent experiments. Asterisk indicates P<0.05 compared with the controls as determined by t test.

**Figure 5:**
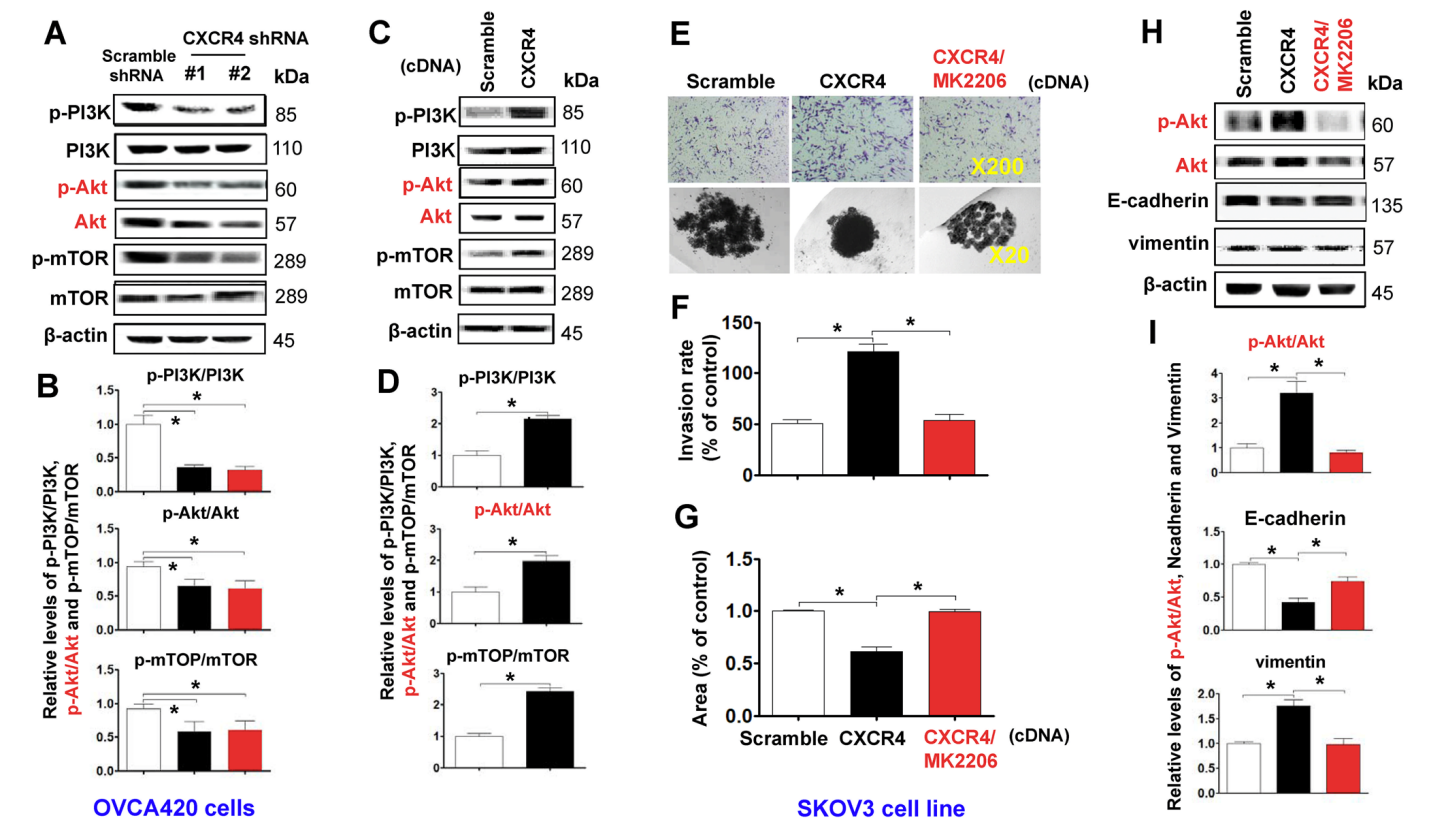
**Characterizing the role of the PI3K/Akt/mTOR pathway in promoting CXCR4 overexpression-mediated ovarian cancer invasion, EMT, and CSC stemness.** PI3K/Akt/mTOR pathway-related protein phosphorylated states indicated were analysed in both CXCR4 shRNA knockdowned-OVCA420 and CXCR4 overexpressed SKOV3 cells by WB with the indicated antibody against each protein, respectively (**A**, **C**). Band density ratios of phosphorylated-PI3K (p-PI3K), -Akt (p-Akt) and -mTOR to total-PI3K (PI3K), -Akt (Akt) and -mTOR (mTOR) were determined by densitometry analysis, respectively (**B**, **D**). Effects of MK-2206 on inhibiting SKOV3 cell invasion induced by CXCR4 overexpressing were analysed by a transwell tumour cell invasion assay (**E**, upper panel). Effects of MK-2206 on inhibiting SKOV3 cell spheroid formation capacity induced by CXCR4 overexpressing were analysed by a spheroid culture in hanging drops assay (**E**, lower panel), which were quantified by counting the total number of cells (invasion rate) from 10 random fields (magnification, 200X) (**F**), and the total spheroid hanging drop area (percentage of control) from the CXCR4-overexpressed SKOV3 culture cell experiments, respectively (**G**). Furthermore, effects of MK-2206 on inhibiting the expression of p-Akt, Akt, EMT-related proteins (E-cadherin, N-cadherin, vimentin and snail) in the CXCR4 overexpressed SKOV3 cells were analysed by WB with the indicated antibody against each protein examined (**H**). Band density ratios of p-Akt to Akt, E-cadherin, N-cadherin and vimentin to β-actin were determined by densitometry analysis, respectively (**I**). Data are presented as the mean ± SD of three independent experiments. Asterisk indicates *P*<0.05 compared with the controls as determined by *t* test. Please note that CSC related protein expression profiles in the CXCR4 overexpressed SKOV3 cell line in the presence or absence of MK-2206 treatment were described in the supplementary materials (Supplementary Figure 5).

