# Correction for: CXCR4 knockdown enhances sensitivity of paclitaxel via the PI3K/Akt/mTOR pathway in ovarian carcinoma

**DOI:** 10.18632/aging.204735

**Published:** 2023-05-15

**Authors:** Dan Zi, Qing Li, Cheng-xiong Xu, Zhi-Wei Zhou, Guan-Bin Song, Cheng-Bin Hu, Fang Wen, Han-Lin Yang, Lei Nie, Xing Zhao, Jun Tan, Shu-Feng Zhou, Zhi-Xu He

**Affiliations:** 1 Department of Obstetrics and Gynecology, Guizhou Provincial People's Hospital, Guiyan, Guizhou, 550002, China; 2 Department of Obstetrics and Gynecology, Affiliated Hospital of Guizhou Medical University, Guiyang, 550004, China; 3 Key Laboratory of Adult Stem Cell Transformation Research, Chinese Academy of Medical Sciences/Stem Cell and Tissue Engineering Research Center, Guizhou Medical University, Guiyang, 550004, China; 4 Key Laboratory of Endemic and Ethnic Diseases and Key Laboratory of Molecular Biology, Ministry of Education, Guizhou Medical University, Guiyang, 550004, China; 5 Department of Pharmaceutical Sciences, College of Pharmacy, University of South Florida, Tampa, FL 33612, USA; 6 Cancer Center, Daping Hospital and Research Institute of Surgery, The Third Military Medical University, Yuzhon, Chongqing, 40042, China; 7 Department of Radiation Oncology, University of Texas Southwestern Medical Center, Dallas, TX 75390, USA; 8 Key Laboratory of Biorheological Science and Technology, Ministry of Education, College of Bioengineering, Chongqing University, Chongqing, 400030, China; 9 Department of Computer Science and Engineering, University of South Florida, Tampa, FL 33620, USA; 10 Department of Bioengineering and Biotechnology, College of Chemical Engineering, Huaqiao University, Xiame, Fujian, 361021, China; 11 Department of Pediatrics, Affiliated Hospital of Zunyi Medical University, Zunyi, 563000, China

**This article has been corrected:** The affiliation of Dr. Shu-Feng Zhou has been clarified. This work was started in Dr. Shu-Feng Zhou's laboratory in 2013, when he was at the University of South Florida. However, he left that position in 2016 and was not affiliated with USF at the time of the article's publication. His current affiliation is the College of Chemical Engineering, Huaqiao University, Xiamen 361021, China. The corrected authors affiliations are listed below.

**Dan Zi^1,2,3,4,5^, Qing Li^6,8^, Cheng-xiong Xu^6,8^, Zhi-Wei Zhou^7^, Guan-Bin Song^7^, Cheng-Bin Hu^9^, Fang Wen^2^, Han-Lin Yang^2^, Lei Nie^2^, Xing Zhao^3^, Jun Tan^4^, Shu-Feng Zhou^5,10^, Zhi-Xu He^3,11^**

^1^Department of Obstetrics and Gynecology, Guizhou Provincial People's Hospital, Guiyang 550002, Guizhou, China

^2^Department of Obstetrics and Gynecology, Affiliated Hospital of Guizhou Medical University, Guiyang 550004, China

^3^Key Laboratory of Adult Stem Cell Transformation Research, Chinese Academy of Medical Sciences/Stem Cell and Tissue Engineering Research Center, Guizhou Medical University, Guiyang 550004, China

^4^Key Laboratory of Endemic and Ethnic Diseases and Key Laboratory of Molecular Biology, Ministry of Education, Guizhou Medical University, Guiyang 550004, China

^5^**Former affiliation:** Department of Pharmaceutical Sciences, College of Pharmacy, University of South Florida, Tampa, FL 33612, USA

^6^Cancer Center, Daping Hospital and Research Institute of Surgery, The Third Military Medical University, Yuzhong 40042, Chongqing, China

^7^Department of Radiation Oncology, University of Texas Southwestern Medical Center, Dallas, TX 75390, USA

^8^Key Laboratory of Biorheological Science and Technology, Ministry of Education, College of Bioengineering, Chongqing University, Chongqing 400030, China

^9^Department of Computer Science and Engineering, University of South Florida, Tampa, FL 33620, USA

^10^**Current affiliation:** Department of Bioengineering and Biotechnology, College of Chemical Engineering, Huaqiao University, Xiamen 361021, Fujian, China

^11^Department of Pediatrics, Affiliated Hospital of Zunyi Medical University, Zunyi 563000, China

