# Supplementary material for: CXCR4 knockdown enhances sensitivity of paclitaxel via the PI3K/Akt/mTOR pathway in ovarian carcinoma
Source: Aging (Albany NY). 2022 Jun 9;14(11):4673–98. doi: 10.18632/aging.203241 (PMC9217704; doi:10.18632/aging.203241)
Supplement: Supplementary Figures [file aging-14-203241-s001.pdf]

SUPPLEMENTARY FIGURES

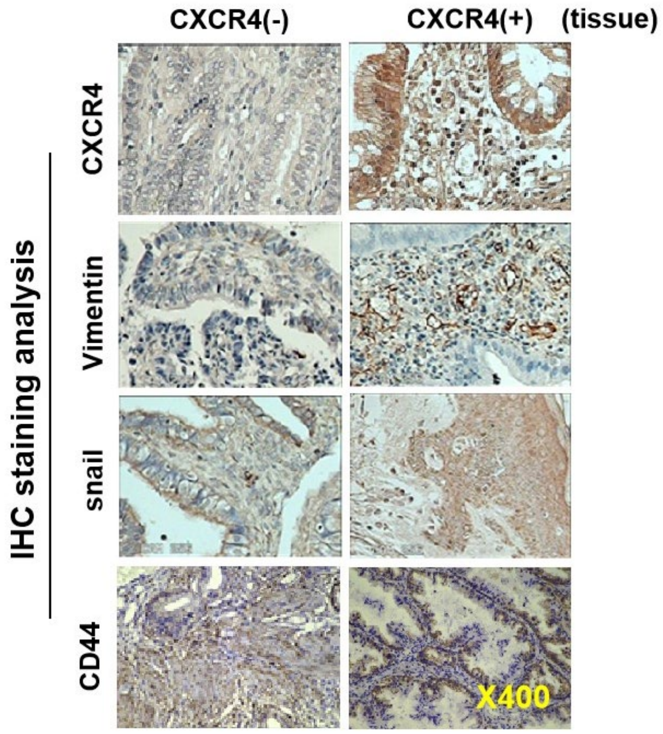

Supplementary Figure 1. Oncopathological examination of CXCR4, EMT- and CSC-related protein expressions in epithelial ovarian cancer and ovarian cancer. Expressions of CXCR4 and vimentin, snail and CD44 in EOC and benign epithelial ovarian tumour tissues were analysed by immunohistochemistry (IHC) staining with the indicated antibody against each protein examined.

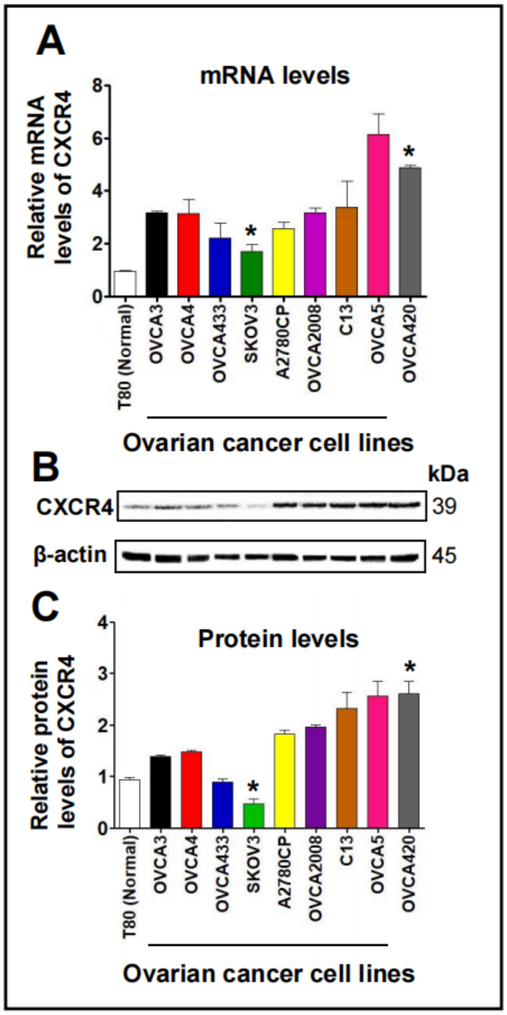

Supplementary Figure 2. Determining CXCR4 expression in a human ovarian surface epithelial cell line and eight ovarian cancer cell lines. The expression of CXCR4 mRNA (**A**) and protein (**B**) levels were analysed by qRT=PCR and WB using an anti-CXCR4 antibody. Band density ratios of each protein examined to β-actin were determined by densitometry analysis as indicated below the WB panel (**C**). Notably, both CXCR4 mRNA and protein levels were highest in OVCAR420 cells and lowest in SKOV3 cells. Data are presented as the mean ± SD of three independent experiments. Asterisk indicates $P < 0.05$ compared with the controls as determined by t test.

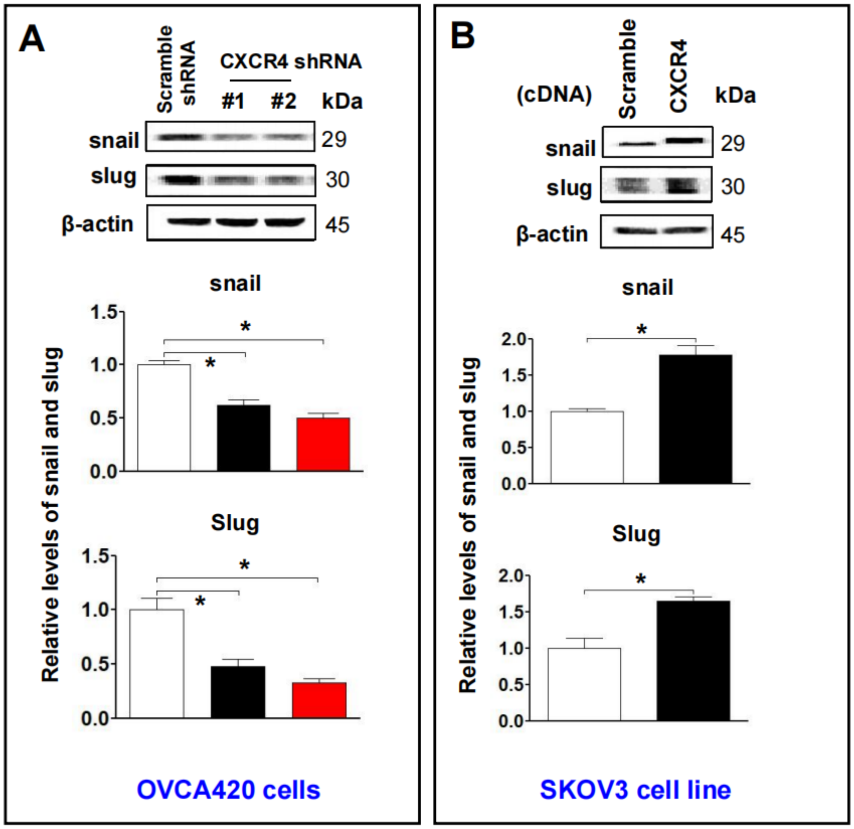

Supplementary Figure 3. Examining effects of CXCR4 knockdown on decreasing the cancer (EOC) invasion capacity. The effects of CXCR4 on the expression of EMT-related snail and slug protein levels indicated in both CXCR4-knockdowned OVCA420 (**A**) and -overexpressed SKOV3 cells (**B**) were analysed by WB with the indicated antibody against each protein examined, respectively. Band density ratios of snail and slug to β-actin in both CXCR4-knockdowned OVCA420 and -overexpressed SKOV3 cell lines were determined by densitometry analysis as indicated below each WB panel. Data are presented as the mean ± SD of three independent experiments. Asterisk indicates $P < 0.05$ compared with the controls as determined by *t* test.

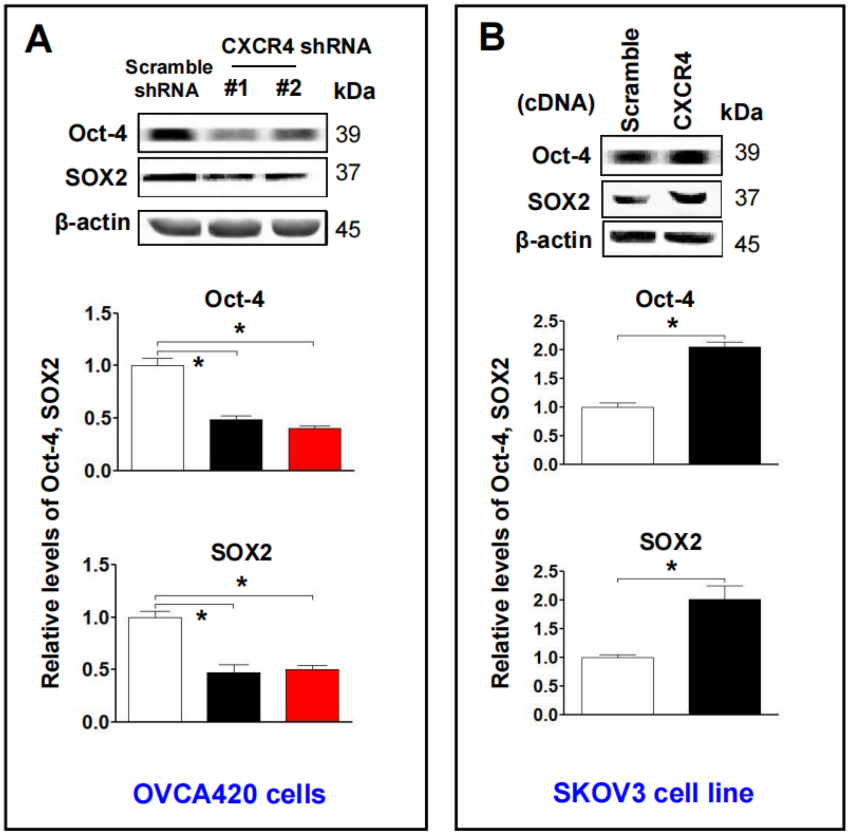

Supplementary Figure 4. Determining effects of CXCR4 overexpression on augmenting the cancer (EOC) spheroid formation capacity, which was markedly opposed by CXCR4 silencing. The effects of overexpression and knockdown of CXCR4 on expression of CSC-related Oct-4 and SOX2 proteins in both OVCA420 (**A**) and SKOV3 (**B**) cells were analysed by WB with the indicated antibody against each protein examined, respectively. Band density ratios of Oct-4 and SOX4 to β-actin were determined by densitometry analysis as indicated below each WB panel. Data are presented as the mean ± SD of three independent experiments. Asterisk indicates P <0.05 compared with the controls as determined by t test.

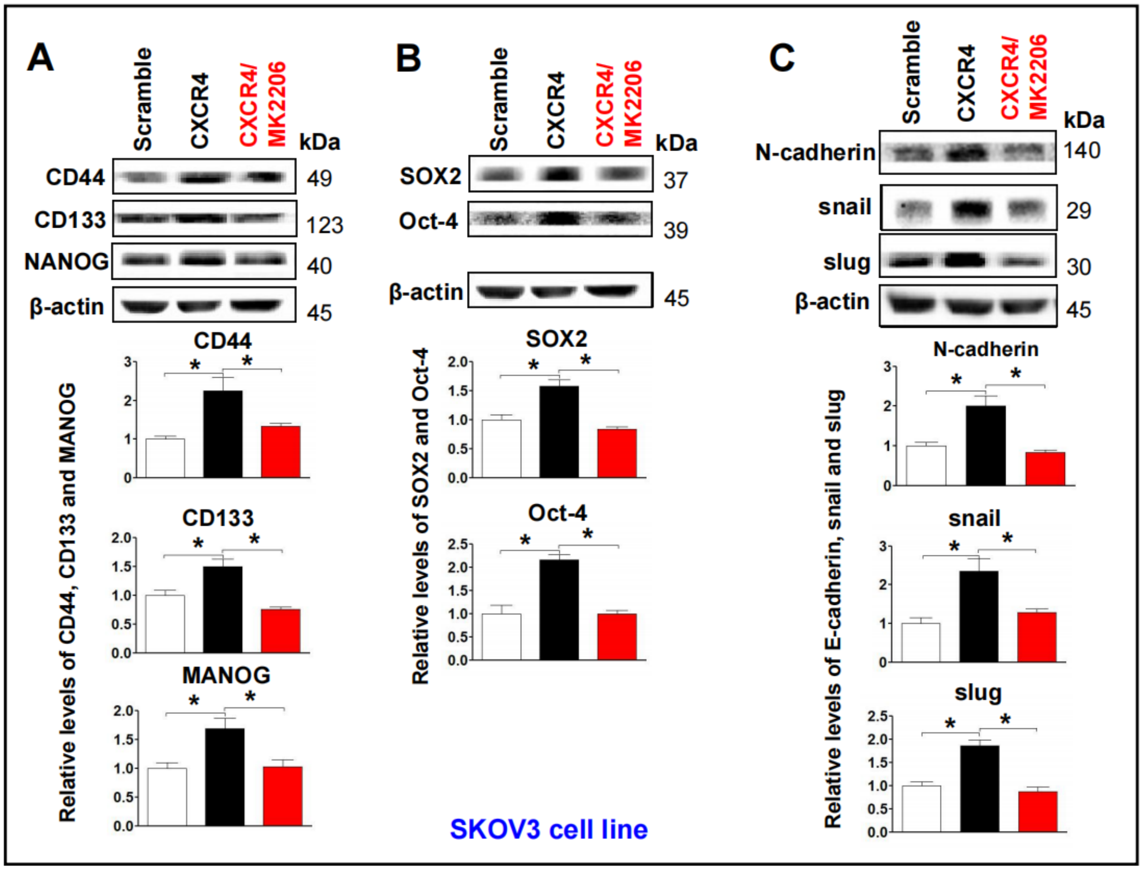

Supplementary Figure 5. Characterizing the role of the canonical PI3K/Akt/mTOR pathway in promoting CXCR4 overexpression-mediated ovarian cancer CSC stemness. The effects of MK-2206 on the expression of stemness-related proteins (CD44, CD133, NANOG, Oct-4 and SOX2) (**A**, **B**), and EMT-related proteins (E-cadherin, snail and slug) in the CXCR4 overexpressed SKOV3 cells (**C**), respectively. Band density ratios of CD44, CD133, NANOG, Oct-4, SOX2, and N-cadherin, snail, slug to β-actin were determined by densitometry analysis as indicated below each WB panel, respectively. Data are presented as the mean ± SD of three independent experiments. Asterisk indicates $P < 0.05$ compared with the controls as determined by *t* test.

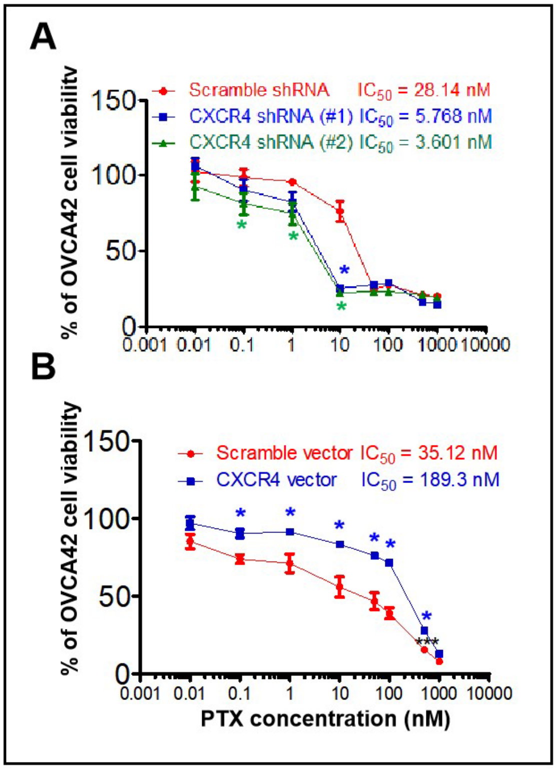

Supplementary Figure 6. Characterizing the effects of CXCR4 knockdown and overexpression on chemosensitivity of OVCA420 and SKOV3 cells to PTX. MTT cytotoxicity assays were used to compare the effects of CXCR4 protein expression on the chemosensitivity of OVCA420 and SKOV3 cells to PTX. CXCR4 knockdown increased the chemosensitivity of OVCA420 cells to PTX (**A**). CXCR4 overexpression decreased the chemosensitivity of SKOV3 cells to PTX (**B**). Data are presented as the mean ± SD of three independent experiments. Asterisk indicates $P < 0.05$ compared with the controls as determined by *t* test.

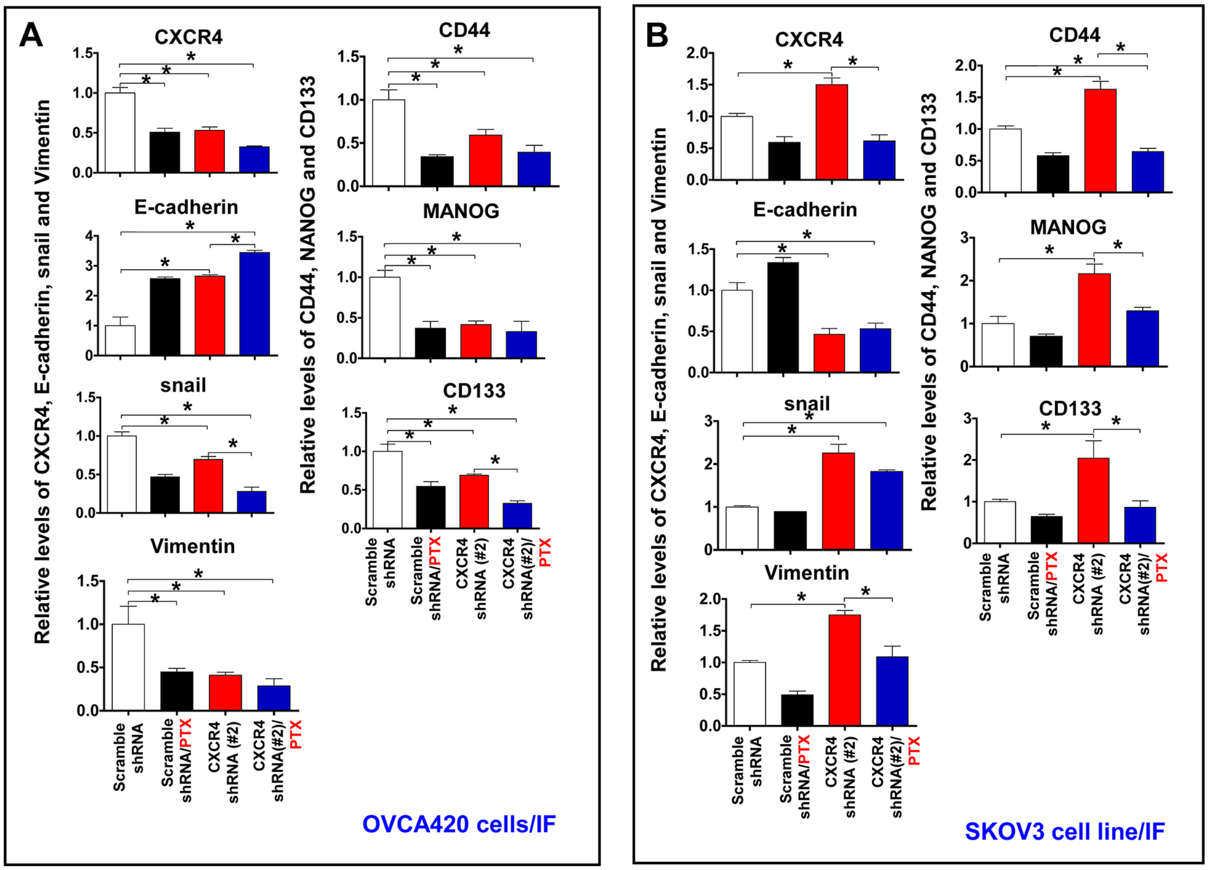

Supplementary Figure 7. Further *ex vivo* IF examining the expression of CXCR4, EMT- and CSC-related proteins in the OVCA420 and SKOV3 cell xenograft tissues from the tumours nude mice following treatment with PTX. IF images were further quantified by counting the total number of staining positive cells for CXCR4, E-cadherin, snail, vimentin, CD44, NANOG and CD133 over controls in both OVCA420 (**A**) and SKOV3 (**B**) tumour cell xenograft mouse tissues from PTX treated nude mice and controls. Data are presented as the mean ± SD of three independent experiments. Asterisk indicates $P < 0.05$ compared with the control as determined by *t* test.
