# Supplementary material for: CXCR4 knockdown enhances sensitivity of paclitaxel via the PI3K/Akt/mTOR pathway in ovarian carcinoma
Source: Aging (Albany NY). 2022 Jun 9;14(11):4673–98. doi: 10.18632/aging.203241 (PMC9217704; doi:10.18632/aging.203241)
Supplement: Supplementary Tables [file aging-14-203241-s002.pdf]

SUPPLEMENTARY TABLES

Supplementary Table 1. The primer information.

Gene name	sequence
CXCR4 shRNA#1	sense: 5′- CGTCCACTTACTGCATCCTCT -3′<br>antisense: 5′- TACTTGTCCGTCATGCTTCTC -3′
CXCR4 shRNA#2	sense: 5′-GCCCTCGATCCCGTACAGCCT-3′<br>antisense: 5′- ATTTCTAGCTTTACGTGATTC -3′
CXCR4 shRNA#3	sense: 5′-GCATTGACCGGAGGAGCAGCA-3′<br>antisense: 5′-ATGCCAGTTAAGAAGATGATG -3′
Scramble shRNA (pLX304-Blast-V5-CXCR4)	sense: 5'-CGCAAATGGGCGGTAGGCGTG-3'<br>antisense: 5'-TACGGGAAGCAATAGCATGA-3'

Supplementary Table 2. The antibodies information for IHC and WB.

Antibody name (company)	Dilution
**Immunohistochemistry**	
CXCR4 (CST)	1:100
E-cadherin (CST)	1:200
N-cadherin (CST)	1:200
Vimentin (CST)	1:200
CD44 (CST)	1:200
CD133 (CST)	1:200
NANOG (CST)	1:200
Oct-4 (CST)	1:200
**Western blotting**	
CXCR4 (Abcam)	1:800
E-cadherin (Santa)	1:1000
N-cadherin (Santa)	1:1000
Vimentin (Santa)	1:1000
CD44 (Santa)	1:1000
CD133 (Santa)	1:1000
NANOG (Santa)	1:1000
Oct-4 (Santa)	1:200
β-catenin (Santa)	1:1000
Akt (Santa)	1:1000
p-Akt at Ser473 (Santa)	1:1000
mTOR (Santa)	1:1000
p-mTOR at Ser248 (Santa)	1:1000
Bcl-2 (Santa)	1:1000
